# Buffer Specificity of Ionizable Lipid Nanoparticle Transfection Efficiency and Bulk Phase Transition

**DOI:** 10.1021/acsnano.4c14098

**Published:** 2025-03-12

**Authors:** Cristina Carucci, Julian Philipp, Judith A. Müller, Akhil Sudarsan, Ekaterina Kostyurina, Clement E. Blanchet, Nadine Schwierz, Drew F. Parsons, Andrea Salis, Joachim O. Rädler

**Affiliations:** † Department of Chemical and Geological Sciences, University of Cagliari & Center for Colloid and Surface Science (CSGI), Cittadella Universitaria, 09042 Monserrato, CA, Italy; ‡ Faculty of Physics, Ludwig-Maximilians University, Geschwister-Scholl-Platz 1, 80539 Munich, Germany; § Institute of Physics, University of Augsburg, 86159 Augsburg, Germany; ∥ European Molecular Biology Laboratory Hamburg Outstation c/o Deutsches Elektronen-Synchrotron, 22607 Hamburg, Germany

**Keywords:** lipid nanoparticles, ionizable lipids, transfection efficiency, DLin-MC3-DMA, pH transition, specific buffer effects

## Abstract

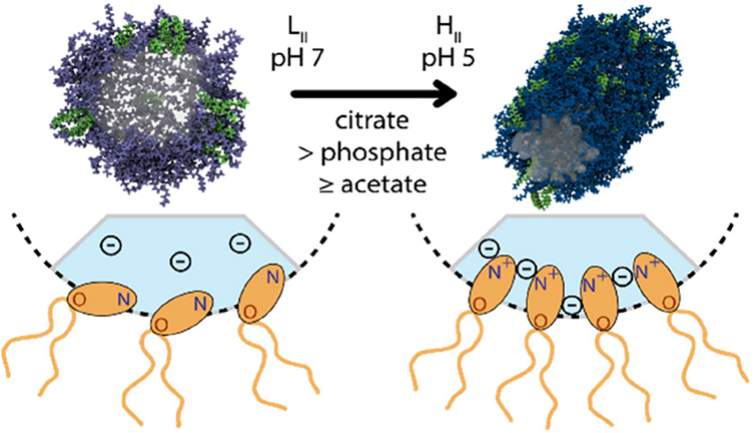

Lipid nanoparticles (LNPs) are efficient and safe carriers for mRNA vaccines based on advanced ionizable lipids. It is understood that the pH-dependent structural transition of the mesoscopic LNP core phase plays a key role in mRNA transfer. However, buffer-specific variations in transfection efficiency remain obscure. Here we analyze the effect of the buffer type on the transfection efficiency of LNPs. We find that LNPs formulated with the cationic ionizable lipids DLin-MC3-DMA (MC3), SM-102, and ALC-315 in citrate compared to phosphate and acetate buffers exhibit earlier onset and stronger mRNA-GFP expression in vitro. Using synchrotron small-angle X-ray scattering (SAXS) we determine the buffer specificity of the pH-dependent structure of ionizable lipid/cholesterol/water mesophases that serve as model systems for the LNP core phase. The results show that the phase transition from inverse micellar to inverse hexagonal with decreasing pH is shifted to a lower transition pH for acetate and phosphate compared with citrate buffer. Based on continuum theory and ion-specific adsorption obtained from all-atom MD simulations, we propose a mechanism for buffer specificity. Citrate stabilizes the inverse hexagonal phase thus shifting the formation of H_II_ to a higher pH. By contrast, phosphate and acetate stabilize L_II_. It stands to reason that the inverse micellar to inverse hexagonal transition, which is facilitated in citrate buffer, enables a sensitized pH response of the LNP core phase. This, in turn, enhances endosomal release efficiency and accounts for the earlier onset of gene expression observed in LNPs prepared with citrate buffer.

Gene delivery carriers have been developed and improved for decades but reached an unprecedented breakthrough through successful, safe, and efficient delivery of mRNA-based vaccination during the COVID-19 pandemic.^1−3^ Specifically, lipid nanoparticle (LNP) formulations have been approved by the Food and Drug Administration (FDA) due to favorable properties such as colloidal stability, low toxicity, and controlled size. While early cationic lipoplexes proved toxic to human cells, LNPs based on ionizable lipids exhibit pH-dependent lipid headgroup ionization and hence a less toxic surface charge. Furthermore, once endocytosed, LNPs overcome endosomal entrapment via endosomal fusion in a pH-dependent manner. LNPs are internalized at physiological pH and experience protonation during the time course of the early endosomal maturation with pH values decreasing to (6.5–5.0).^4,5^ The process of charging ionizable lipid headgroups facilitates endosomal fusion, releasing mRNA into the cytosol. The exact process of how LNPs fuse with the endosome membrane is the subject of intense research. In cationic lipid-based lipofection, it has been rationalized that the mesoscopic bulk structure of lipoplexes affects fusogenicity, with the inverse hexagonal phase^6^ and cubic phase^7^ being particularly fusogenic compared to lamellar internal packing. LNPs are nanoscale particles with a well-defined surface and core composition. The interior of LNPs consists of a cationic ionizable lipid/cholesterol moiety complexed with mRNA. The surface is composed of a monolayer that includes all lipid components, specifically the stabilizing PEG-lipid/DSPC, and cholesterol. The core of the LNP, structured by ionizable lipids, exhibits pH-dependent mesostructures, leading to multiple phases and structural transitions as the pH decreases.^8−10^ The behavior of the core phase has been studied using binary ionizable lipid/cholesterol bulk phases as model systems.^9^ This simplification to just two lipid components is feasible because both DSPC and PEG are largely absent from the LNP core.

It is generally understood that lyotropic mesophases depend on the lipid chain splay described by the Israelachvili shape factor.^11,12^ Ionizable lipids with strongly conic shape form inverted phases, which with increasing headgroups size range from inverse micellar disordered, L_II_, to disconnected inverse micellar cubic, I_II_, to inverse hexagonal, H_II_, and bicontinuous cubic phases, Q_II_. Thereby the (I_II_–H_II_) as well as (H_II_–Q_II_) transitions change the connectivity of the lipid network.^13^ In recent work, we showed that specifically, the ionizable lipid MC3 shows a structural phase transition with decreasing pH from inverse micellar cubic phase with space group *Fd*3*m* to inverse hexagonal (denoted *Fd*3*m*-H_II_ transition in the following).^14^ X-ray scattering from full LNPs also provides evidence that a similar structural transition occurs within the LNPs as a function of pH, assuming that the LNP core mesophase is predominantly formed by ionizable excess lipid and cholesterol. The pH-dependent lipid core transition has been suggested as a critical factor in inducing endosomal fusion and hence mRNA escape efficiency.^14^ Therefore, further investigation of ionizable lipid/cholesterol bulk phases as LNP core mimics is valuable for gaining insight into the LNP-endosomal fusion mechanism.

The critical pH value of the structural transition coincides with the p*K*_a_ value of the ionizable lipid. The regulation of pH in both chemical and biological systems is carried out by buffers, a mixture of a weak acid (HA) or base (B) with its conjugate base (A^–^) or acid (BH^+^). According to the Henderson–Hasselbalch equation, the only important parameters for the choice of buffer are the p*K*_a_ and concentrations of the buffer components. Little attention has been paid to the chemical identity of the weak electrolytes and their conjugate species used to prepare the buffer.^15^ However, starting from the pioneering work by Ninham and co-workers on restriction enzyme activities,^16^ several studies have shown that the chemical nature of the buffer, even at the same nominal pH, can have important unexpected effects on the investigated biosystem. For example, buffers have been found to affect specifically the behavior of proteins, including lysozyme electrophoretic mobility^17^ and adsorption,^18^ Brownian motion of BSA,^19^ and more recently, DNA thermal stability,^20^ DNA interactions with lipid bilayers,^21^ and the formation of a protein corona around nanoparticles.^22^ In fact, “specific buffer effects” can be included in the wider classification of “ion-specific effects” first observed by Hofmeister in 1888.^23^ The “Hofmeister series” is an order based on ion-induced protein precipitation (salting out) or solubilization (salting in). A conventional explanation of the Hofmeister series was proposed^24,25^ invoking Jones and Dole’s work^26^ on the viscosity of aqueous salt solutions. Ions were classified as “kosmotropic” (order maker) or chaotropic (disorder maker) on the basis of their interaction with water quantified through the value (and sign) of the Jones–Dole viscosity B coefficient. More recent theoretical and simulation work explains ion specificity as the result of a delicate interplay between electrostatic, hydration, and ion-dispersion forces.^27,28^ Whatever the details of the mechanisms explaining “Hofmeister phenomena”, it must be considered that ions play an important role in modulating biological mechanisms in a way that is not fully understood. For example, Meulewaeter et al.^29^ found that Tris and HEPES buffers improved cryoprotection and, more importantly, the transfection efficiency of mRNA-LNPs compared to PBS buffer. What emerges from previous studies is that the mechanism of transfection, based on bulk phase transitions, is controlled not only by pH but is also specifically affected by the buffer ions used to control pH.^30,31^

The present work aims to investigate the effect of the choice of preparation buffer solution (here, citrate, phosphate, and acetate buffers) on the transfection efficiency of LNPs formulated with the ionizable lipid DLin-MC3-DMA (MC3), SM-102, and ALC-315, in combination with cholesterol, DSPC, and DSPE-PEG2000, respectively. In order to explain the observed buffer-specific transfection efficiency, we investigated the corresponding bulk phase composed of ionizable lipid/cholesterol model systems as a function of pH in different buffers using synchrotron small-angle X-ray scattering (SAXS). We present a mechanism for the observed buffer-specific pH shift of the inverse micellar to inverse hexagonal bulk phase transition in terms of changes in the area per lipid headgroup affected by pH and by ion-specific adsorption based on all-atom MD simulations. The study highlights the role of buffer ions in the pH-dependent structural transitions in ionizable lipid bulk phases. We propose that a similar pH-dependent structural transition might occur in the excess lipid region within the LNP core phase, facilitating endosomal release and hence resulting in an earlier gene expression onset compared to phosphate and acetate buffer.

## Results and Discussion

### Specific Buffer Effects on MRNA LNPs and Transfection Efficiency

**Figure 1:**
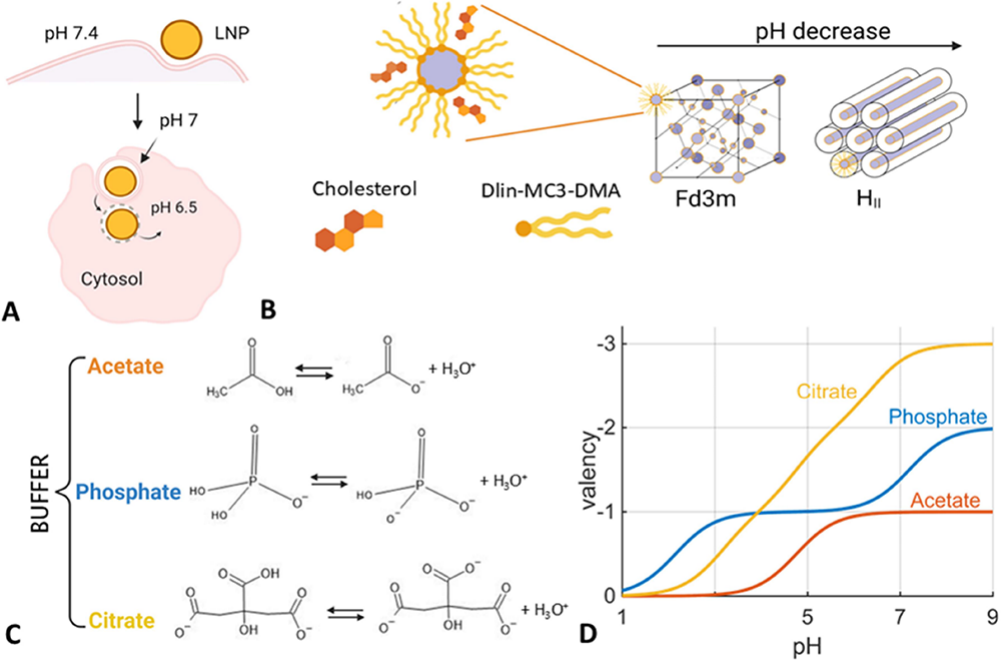
Buffer specificity of LNP-mediated mRNA delivery. (A) Schematic drawing of internalization via endocytosis. Endosomal release of LNPs occurs as a consequence of acidification inside the endosome from pH 7 to about pH 6.5. (B) Schematic drawing of the bulk phase made by ionizable lipid DLin-MC3-DMA and cholesterol together with the bulk phase transition from *Fd*3*m* to H_II_ as pH decreases. (C) Buffer used during the dialysis process is chosen among a range of buffers: citrate (p*K*_a_ 6.4), phosphate (p*K*_a_ 7.2), and acetate (p*K*_a_ 4.8). To evaluate the phase transition, a wide range of pH values from 3.5 to 7.0 in a 0.5 pH unit has been studied. (D) Buffer valency at each pH depending on p*K*_a_ values.

LNPs are composed of an external layer composed of PEG polymer with DSPC and MC3 lipids with an internal bulk phase composed of DLin-MC3-DMA and cholesterol. MC3 is an ionizable lipid with a p*K*_a_ of 6.44,^32^ which makes it pH-sensitive (Figure 1A,B). The LNPs are internalized inside living cells at pH ∼ 7 until reaching the endosome, where mRNA is released at pH ∼ 6–6.5 (Figure 1A). To study the buffer effects, three common buffers were chosen (Figure 1C,D).

According to the Henderson–Hasselbalch equation (pH = p*K*_a_ + log[A^−^]/[HA]), citrate, phosphate, and acetate with p*K*_a_ of 6.40, 7.22, and 4.80, respectively, cover the whole pH range of the LNP route from internalization (pH 7) to mRNA release (pH 6). Sodium citrate buffer has been used in cationic lipid design for siRNA delivery^1^ and is by far the most used for LNP preparation at acidic pH. Sodium phosphate buffer was used with KCl and NaCl to store Pfizer’s mRNA vaccine.^30^ Sodium acetate buffer has been used to dilute mRNA for LNP encapsulation^33^ and as a dialysis buffer for ethanol removal after LNP preparation.^34^

**Figure 2:**
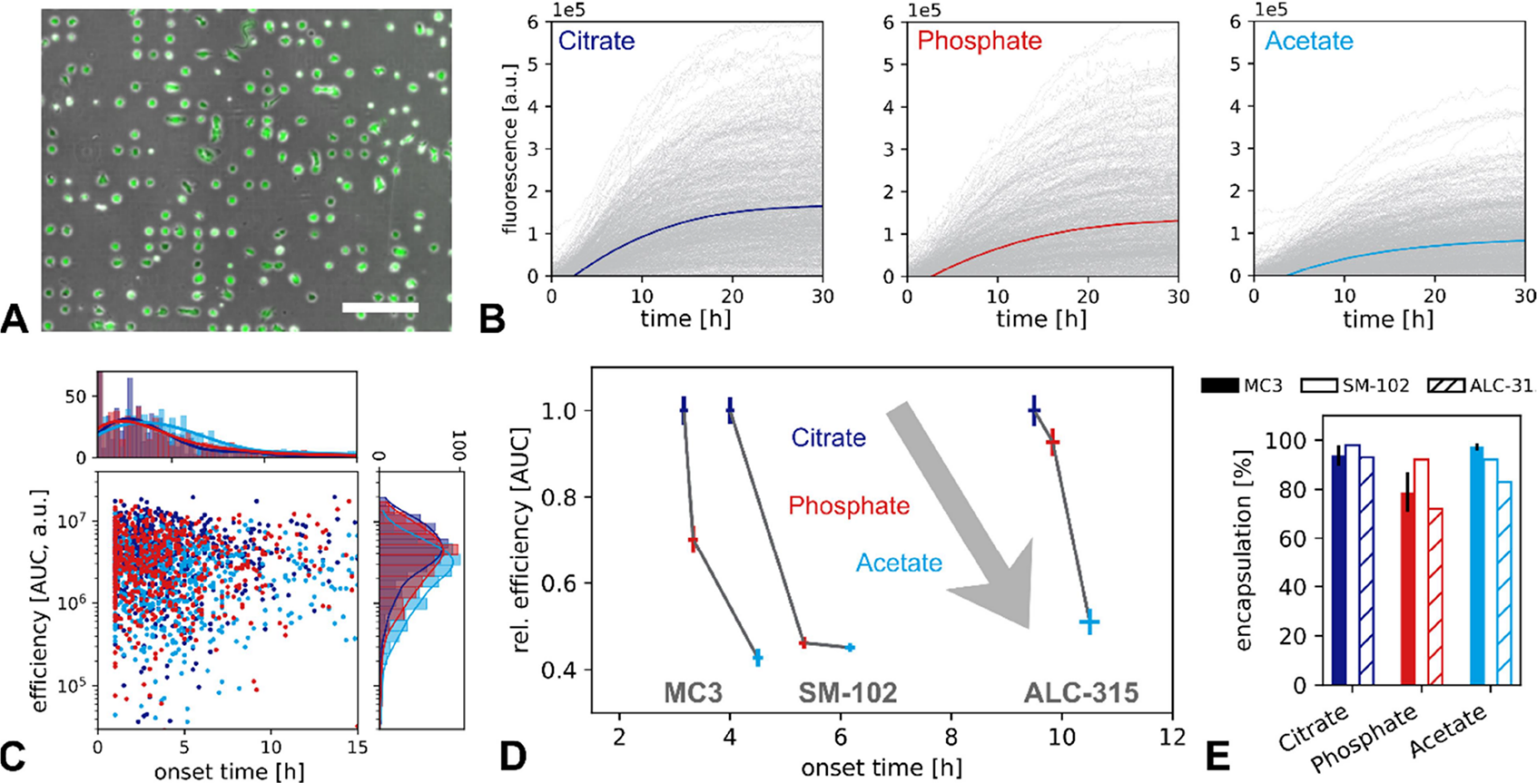
Single cell transfection experiments: (A) Culturing cells on microfabricated single-cell arrays allows recording of hundreds of single-cell fluorescence trajectories in parallel. (B) Single cell expression time courses of eGFP-mRNA/LNPs (gray lines) show different fluorescence kinetics for different preparation buffers (averaged time courses shown in color). (C) Scatter plot of single cell expression efficiencies, area under the curve (AUC), versus expression onset times. (D) Relative efficiency in terms of AUC relative to efficiency of the LNP prepared in citrate buffer versus onset time of fluorescence. The three LNP buffer conditions exhibit an apparent Hofmeister ordering, showing earlier onset and higher protein expression for citrate compared with phosphate and acetate. Error bars indicate the standard error of the mean. (E) Encapsulation efficiencies for LNPs prepared using three different buffers and three different ionizable lipids.

To investigate buffer-specific effects on transfection kinetics, we prepared eGFP-mRNA LNPs in citrate, phosphate, or acetate buffer as described previously,^14^ followed by dialysis into water. We performed live-cell imaging on single-cell arrays (LISCA) after transfection and measured protein expression in a time-resolved manner.^35^ To this end, LNPs were preincubated in a cell culture medium supplemented with serum and transfected into the human liver carcinoma cell line (HuH7) seeded on a single-cell slide (Figure 2A).

Observation of the GFP expression kinetics of single cells resulted in a fluorescence trajectory for each cell (Figure 2B). Distinct differences between the buffers were apparent from these traces. Averaging all traces revealed the highest overall GFP level for LNPs prepared in citrate buffer followed by those prepared in phosphate buffer and the lowest expression from the acetate LNPs. The single-cell resolution allowed for the calculation of the total protein amount per cell expressed as the area under the curve (AUC) and the distinct onset of protein expression for every single cell (Figure 2C). Protein expression varied depending on the buffer in which the LNP was prepared, with the highest eGFP expression for the citrate, followed by phosphate, and lowest for the acetate buffer. Figure 2D shows the mean AUCs, in relative units normalized to the AUC in citrate, for all three ionizable lipids versus the mean expression onset. The buffer-specific effect appears to follow a conventional Hofmeister series with citrate > phosphate > acetate for the expression. The observed ordering in the onset times exhibits an inverse relation between the expression efficiency and expression onset time (Figure 2D). This confirms a correlation between fast onset and high protein expression levels as previously reported in time-resolved studies.^36^ To rule out buffer-specific effects on LNP preparation, LNP encapsulation (Figure 2E) and particle size (Table S1) were measured as controls. Note the encapsulation efficiencies and sizes are not affected by buffers within the accuracy of the measurement. In the case of MC3 the eGFP-mRNA LNPs encapsulation efficiency in buffer, 50 mM at pH 3, were (94 ± 4)% in citrate, (79 ± 8)% in phosphate and (97 ± 2)% in acetate respectively.

In summary, these findings indicate that the underlying endosomal release mechanism but not the formulation is dependent on the LNP preparation buffer.

### Specific Buffer Effects on Bulk Phase Transitions

**Figure 3:**
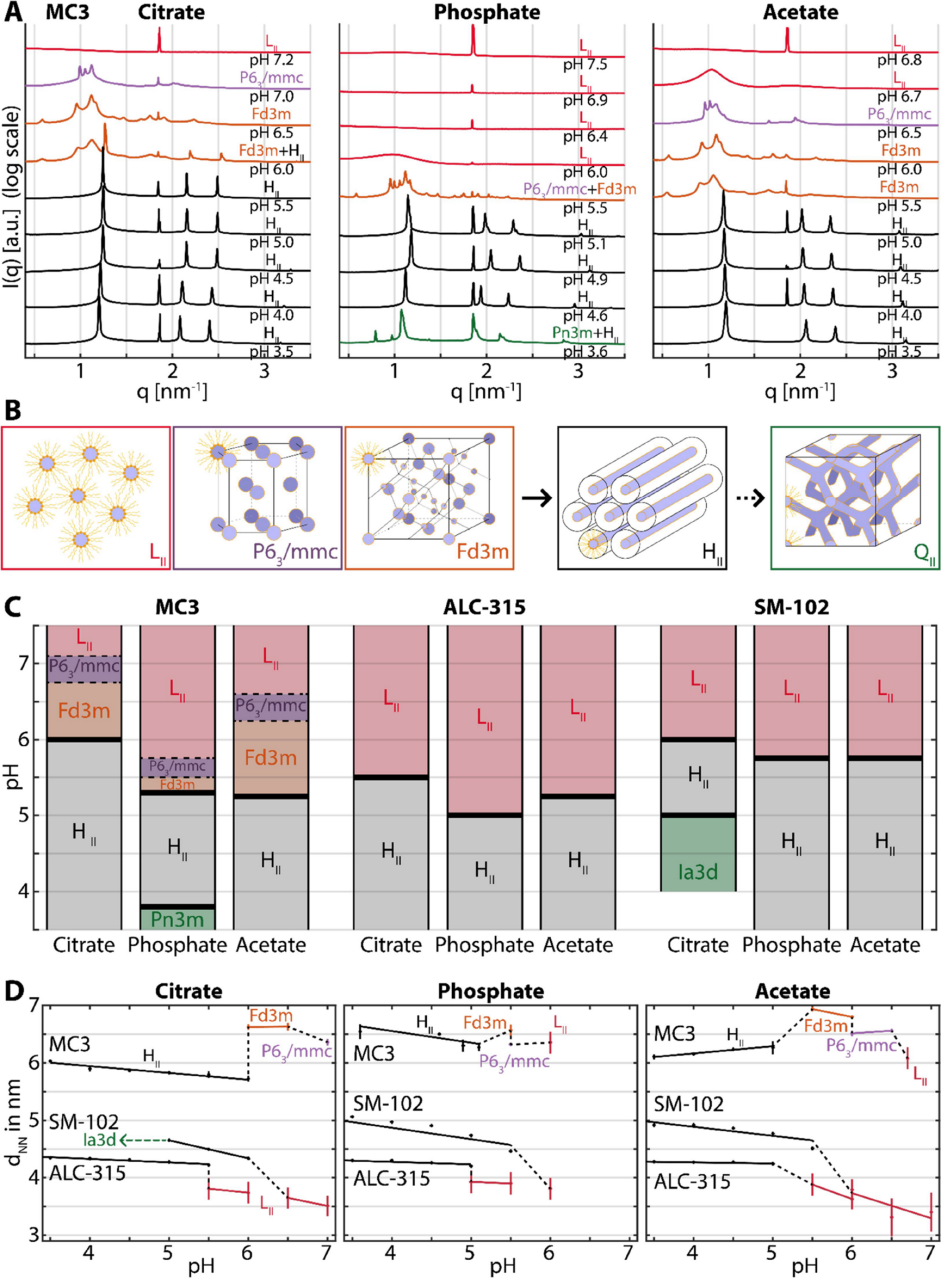
Buffer specific effects on ionizable lipid mesophases. (A) SAXS measurements of MC3-cholesterol bulk samples dialyzed in the presence of 50 mM citrate, acetate, and phosphate buffer and NaCl 150 mM across a pH range of approximately 3.5 to 7.5. (B) Sequence of lipid phase symmetries with increasing protonation showing the order L_II_, *P*6_3_/*mcc*, *Fd*3*m*, H_II_ and Q_II_. (C) Phase diagrams showing the buffer-specific pH dependence for MC3, ALC-315, and SM-102. (D) Nearest neighbor distance *d*_NN_ between centers of encapsulated water micelles/tubes for MC3, ALC-315, and SM-102 with H_II_, *Fd*3*m*, *P*6_3_/*mcc*, and L_II_ phases as a function of pH for the three different buffers.

Next, we investigate the impact of buffers on the phase behavior of ionizable lipid/cholesterol bulk phases as a function of pH. These two-component bulk phases serve as a model for the inner core structure of LNPs, given that PEG and DSPC are restricted to the outer shell of the LNPs. As described in the methods section, the preparation of bulk phase samples consists of three dialysis steps to mimic LNP production by replacing the ethanol with buffer. The buffer (citrate, phosphate, or acetate) at the pH of choice comes into play in the third dialysis step. Figure 3A presents the SAXS scattering profiles for MC3/cholesterol/buffer phases in the presence of citrate, phosphate, and acetate buffers. In the following sections, we describe the pH-dependent structural transitions observed. As shown in Figure 3B decreasing the pH induces a phase transition from inverse micelles (L_II_)^37^ to inverse hexagonal phase (H_II_).^37^ The inverse micellar phase undergoes ordering transitions from a disorder L_II_ through close-packed *P*6_3_/*mcc*^37,38^ toward an inverse cubic *Fd*3*m* phase.^39^ This order of inverse phases as a function of pH has been reported before for MC3/cholesterol in citrate buffer.^10^ Here we find that the same behavior is also found in phosphate and acetate buffer but with slightly shifted pH values of transitions. In citrate buffer, *P*6_3_/*mmc* and *F*d*3m* phases are formed at pH 7.0 and pH 6.5, respectively, with decreasing pH. The same transitions are observed in the presence of acetate, but at a lower pH (*P*6_3_/*mmc* at pH 6.5 and *Fd*3*m* at pH 6.0). A bigger difference is observed for phosphate, resulting in the L_II_ phase being found in the region from pH 7.5 to 6.0 and a mixed phase *P*6_3_/*mmc* + *Fd*3*m* at pH 5.5.

This inverse cubic-to-inverse hexagonal (*Fd*3*m*–H_II_) transition occurs at pH= 6.0 for citrate and at pH = 5.0 for acetate and phosphate (Figure 3A–C). Remarkably the critical pH value is shifted by one pH unit for acetate and phosphate compared to that for citrate. To demonstrate that the transition from inverse micellar (L_II_) to inverse hexagonal phase (H_II_) is a universal characteristic of clinically relevant ionizable lipids we also present the phase behavior of ALC-315/cholesterol and SM-103/cholesterol for all three buffers. We observe that the specific buffer effect on the L_II_–H_II_ transition follows the trend pH (citrate) > pH (phosphate) = pH (acetate). This behavior can be explained by the common conic lipid shape factor which promotes inverse micellar phases at neutral pH.

### Nearest Neighbor Distance (*d*_NN_)

The dimensions of the bulk phase are characterized by the nearest neighbor distance (*d*_NN_), determined by extracting the lattice constants from SAXS measurements (Table S3). *d*_NN_ is defined as the smallest distance between neighboring water cores or channels. Significant differences in *d*_NN_ as a function of pH are seen in Figure 3D. We observe a general trend of increasing *d*_NN_ with a decrease in pH throughout all lipids and buffers. This general trend is caused by the increasing charge at the ionizable lipid’s headgroup with decreasing pH. As described later in the theoretical section increasing electrostatic repulsion reduces the curvature of the lipid–water interface and causes an increasing lattice spacing. We find two distinct exceptions to this rule in the data. The first exception to this trend is the transition from *Fd*3*m* to H_II_ which is accompanied by a reduction of *d*_NN_. This discontinuity is due to the packing symmetry of H_II_ in contrast to the face-centered cubic *Fd*3*m* symmetry. We will come back to the associated energies in the inverse micellar to inverse hexagonal transition in the section on the mechanism of the pH transition. The second exception is the anomalous behavior found in the case of the H_II_ phases of MC3 in acetate buffer, which appears to shrink with increasing protonation.

### Effect of Ionic Strength and Temperature on the Bulk Phase

Charging of the lipid head groups, and consequently, the geometric packing parameter of the lipids is influenced by pH, temperature, and salt concentration. In the following, we examine these parameters in detail in the case of MC3 lipid. The previous experiments were carried out by using a fixed concentration of 50 mM for all buffers at different pH values. However, the charge (*z*) and the concentration (*c*) ratio between the acidic and the basic components of the buffer depends on pH (Figure 1C,D). For instance, at pH 5.5, the ionic strength for the different 50 mM buffers is 151 mM for citrate, 52 mM for phosphate, and 42 mM for acetate. To understand if the observed buffer specificity (Figure 2) could be ascribed to an ionic strength effect of the different 50 mM buffers, SAXS measurements were performed of the LNP bulk phases dialyzed in the presence of citrate and acetate at pH 5.5 and at a defined ionic strength (namely 160, 200, and 300 mM). pH 5.5 was chosen since all buffers showed a clearly ordered structure (*Fd*3*m* or H_II_). Figure S1A shows that, for citrate buffer, H_II_ is the only phase occurring at pH 5.5 for the three ionic strengths. In the case of acetate (Figure S1B), *Fd*3*m* and H_II_ phases coexist at all ionic strengths, but an increase in ionic strength results in an increase in the signal of the inverse hexagonal structure compared to the *Fd*3*m* phase. The results indicate that electrostatic interactions play a role in driving the pH-dependent structural transition. It is known for triethanolamine buffer that an increase of ionic strength results in a shift in the equilibrium of −NH^+^ ⇌N + H^+^ toward the left, corresponding to a shift to higher effective pH (that is lower H^+^ activity).^40^ Similarly, in our system, an increase in ionic strength would enhance the protonation of the MC3 headgroup which would stabilize H_II_ with respect to the *Fd*3*m* phase. We also investigated the effect of temperature at 22 and 37°C under pH 5.5. It is important to examine whether the transitions of the inner bulk phase of LNPs, which we consider the driving mechanism of mRNA transfection, occur in the same pH range observed at lab temperature compared to body temperature. In Figure S1B, we find the temperature increase favors the phase transition from inverse hexagonal H_II_ to *Fd*3*m* + H_II_ in the case of citrate and from *Fd*3*m* + H_II_ to *Fd*3*m* for acetate. That is, the temperature increase has the opposite effect of an increase in ionic strength (Figure S1A), tending to stabilize F3dm and shifting the *Fd*3*m*-H_II_ transition to lower pH values. The temperature dependence is consistent with the trend we would expect from the van’t Hoff equation for the temperature dependence of an acid equilibrium.^41^ The −NH^+^ ⇌N + H^+^ equilibrium is pushed toward dissociation at higher temperatures, leaving the MC3 molecule uncharged. This can be interpreted as an equivalent to pushing the system toward the behavior that would be expected at a lower pH. In summary, the effect of increasing ionic strength is to favor the inverse hexagonal phase, i.e., shift the phase transition to higher pH values, while that of increasing temperature is to favor the inverse micellar phases, i.e. pushing the phase transition to lower pH.

### Mechanism of pH Transition and Buffer Specificity

**Figure 4:**
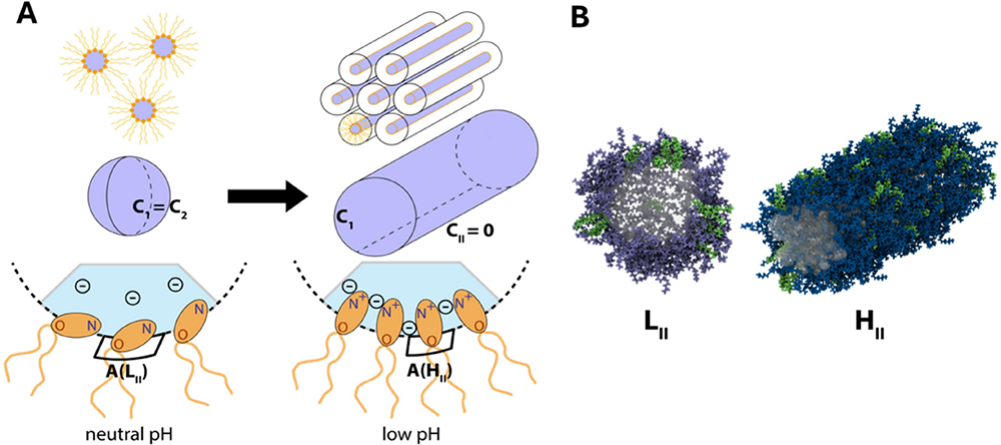
(A) Schematic drawing of the lipid conformation and geometric curvature in the inverse micellar (L_II_) and inverse hexagonal (H_II_) phase. At low pH, the lipid headgroups become protonated and extend outward, reducing the area per headgroup. (B) Snapshots of MD simulations of the L_II_ and H_II_ phases of the MC3/cholesterol system. The area per lipid obtained from MD simulations is *A*(L_II_) = 0.53 nm^2^ and *A*(H_II_) = 0.45 nm^2^. The protonated and neutral MC3 lipids are represented by dark and light blue colors respectively, cholesterol is green, and water is shown in transparent gray.

In the following, we discuss the mechanism that drives the pH-dependent L_II_–H_II_ transition in lipid mesophases. We then ask the question of why this mechanism is buffer-specific. Transfection efficiency measurements (Figure 2D) showed a buffer-specific effect that follows a (conventional) Hofmeister series with citrate > phosphate > acetate. In SAXS measurements, this ordering is also observed for the pH value of the MC3 bulk phase transitions *Fd*3*m–*H_II_. The bulk phase transitions observed by changing pH and ionic strength involve the MC3 charge, but crucially also involve a change in the curvature and area per MC3 headgroup. The balance between these properties determines the phase transition pH. Then, buffer specificity introduces shifts to that transition pH via the specific adsorption of buffer ions at the water–lipid interface (Figure 4A). The formation of the charge on MC3 at low pH favors the H_II_ phase over the inverse micelle phases due to the higher mean curvature of the latter, which gives the inverse micelle phases a larger positive electrostatic energy. But at the same time, the curvature of the interface and area per headgroup affects the L_II_–H_II_ transition via the elastic bending energy of the lipid layer,^28^ favoring the inverse micelle phases over the inverse hexagonal phases.

In general, energy is required if the lipid monolayer bends away from its preferred curvature. The bending energy is characterized by bending moduli κ and κ_G_, and may be quantified via a Helfrich bending energy, *E*_bend_ = κ(*C*_1_ + *C*_2_ – *C*_0_)^2^/2 + κ_G_*C*_1_*C*_2_, where *C*_1_ = 1/*R*_1_ and *C*_2_ = 1/*R*_2_ are the curvatures associated with the radii *R*_1_ and *R*_2_ in perpendicular directions along the surface of the lipid layer layer and *C*_0_ is the so-called spontaneous total curvature due to the conical lipid shape. Although spherical inverse micelles with *C*_1_ = *C*_2_ have lower individual curvatures (larger radii, corresponding to larger *d*_NN_ values in the case of MC3), the cylinders of the inverse hexagonal phase have a lower total curvature (*C*_1_ + *C*_2_) because of the flat dimension along the axis of the cylinders with *C*_2_ = 0 (see also cartoon in Figure 4A). Protonation of the ionizable lipid headgroup will decrease the spontaneous curvature and drive the system from L_II_ into the H_II_ phase. The exact transition point depends on the ratio κ_G_/κ, i.e., the contribution of Gaussian curvature and mean curvature to the total bending energy.^42,43^ The electrostatic energy due to the headgroup charge favors the formation of the inverse hexagonal phase at low pH, while the higher spontaneous curvature favors the formation of the inverse micelle phases at high pH. The balance between the two explains the broad trend of the MC3 phase transitions, with the transition point lying at a pH close to the MC3 p*K*_a_ inside the LNPs of 6.44.^44^

**Figure 5:**
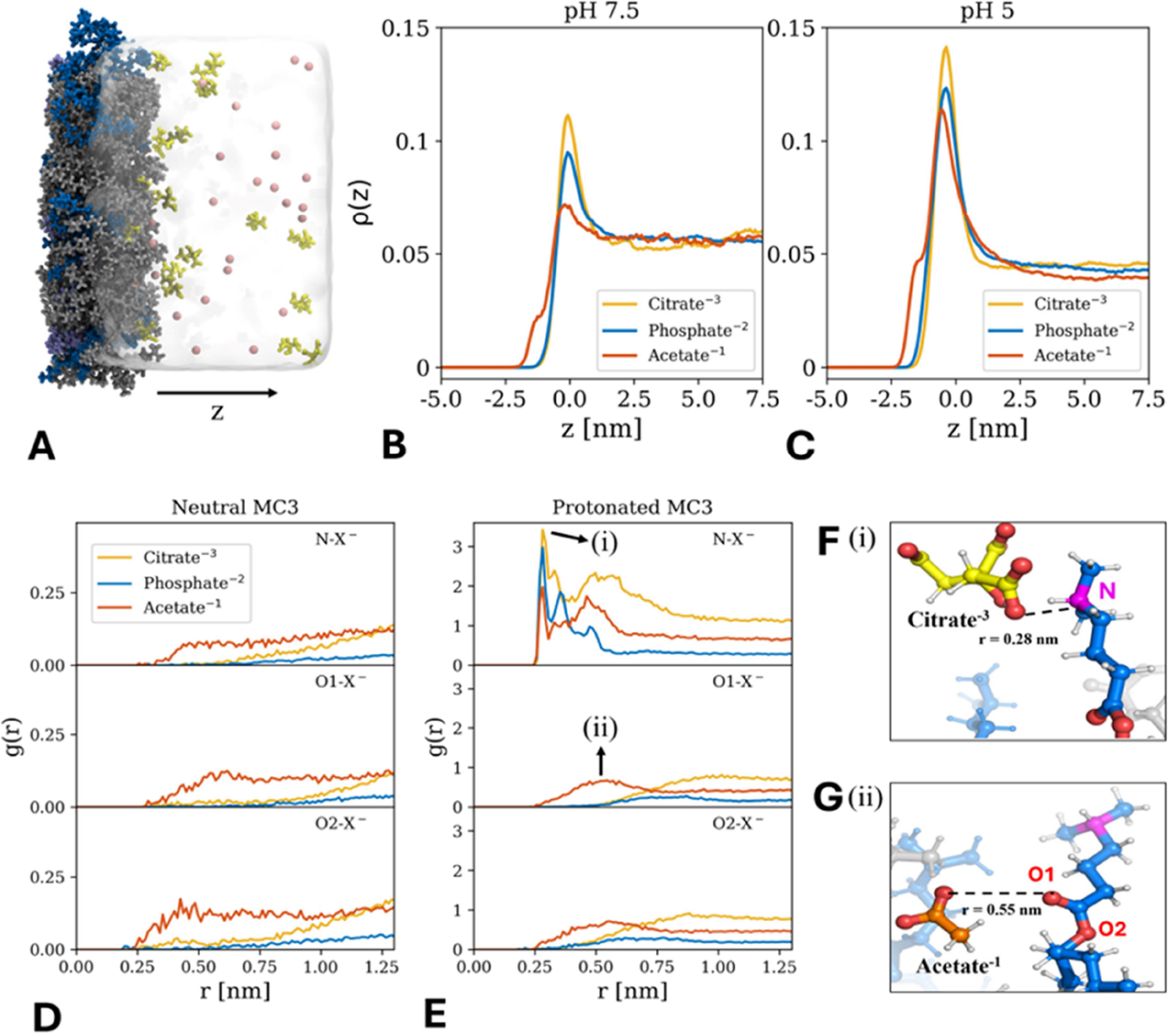
(A) Simulation snapshot of the lipid–water interface of the monolayer system at pH 5. Charged and uncharged MC3 lipids are shown in dark and light blue, respectively. POPC is shown in gray, citrate ions in yellow, and sodium in pink. (B, C) Normalized probability distributions of the buffer ions for pH 7.5 and pH 5 along the *z* axis (perpendicular to the interface as indicated in A). (D, E) Radial distribution function g(r) of the different buffer ions around the nitrogen (N) and oxygen (O1, O2) atoms of an uncharged or charged MC3 molecule in the monolayer at pH 5. (F, G) Selected simulation snapshot of citrate and acetate at distances indicated by the arrows in panel E.

To explain the buffer-specific order observed for bulk phase transitions, we propose a mechanism of specific ion adsorption with a consequent buffer-specific change in the area per headgroup. As illustrated in Figure 4A, we identify near (charged N group) and far (O ester group) moieties within the headgroup. The N-moiety remains in contact with the aqueous phase, whereas the O-moiety may not be in contact, depending on the headgroup orientation (Figure 4A). The strength of interactions of buffer ions with the lipid surface, including the N-moiety, follows the conventional series citrate > phosphate > acetate, confirmed by our MD simulations (Figure 5B). On the other hand, MD simulations (Figure 5C), and evaluation of London dispersion coefficients of ions (Table S4) with the O-moiety, suggest that adsorption at the O-moiety would be stronger for acetate than other buffer ions. Adsorption of acetate at the O-moiety requires the ion to penetrate deeper into the lipid phase and, therefore, is expected to lead to an increase in the area per headgroup. In this way, acetate stabilizes L_II_, consistent with SAXS data. At the same time, the adsorption of negatively charged ions at the N-moiety is expected to reduce the repulsion between the positively charged MC3 headgroup and will therefore reduce the area per headgroup at low pH. Consequently, citrate anions with preferred adsorption at the (positively charged) N-moiety (Figure 5B) decrease the area per headgroup, increasing the bending modulus and shifting the *Fd*3*m*-H_II_ transition to a higher pH by stabilizing H_II_. Acetate ions with preferred adsorption at the O-moiety, likely involving London dispersion forces, increase the area per headgroup, decreasing the bending modulus and shifting the *Fd*3*m*-H_II_ transition to a lower pH by stabilizing *Fd*3*m*. This mechanism, involving two types of specific interactions between the buffer ions and the lipid headgroups, in particular, with the positively charged N-moiety (via electrostatic interactions) and the neutral O-moiety (via nonelectrostatic dispersion forces), is in agreement with the results obtained by investigating the effect of ionic strength. Indeed, results in Figure S1, showing different SAXS patterns for the same ionic strengths obtained with citrate and acetate, confirm the occurrence of different mechanisms for the two buffers. The inverse hexagonal H_II_ phase observed for citrate at the three investigated ionic strengths suggests its preferential interaction with the positively charged MC3 headgroups. By contrast, the coexisting *Fd*3*m* and H_II_ phases, observed for acetate at the three ionic strengths, are consistent with a partial stabilization of the neutral form of the MC3 headgroup. In short, we can identify a direct buffer effect mediated via electrostatics and ion dispersion interactions and an indirect effect via a consequent change in the area per headgroup.

### All-Atom MD Simulations of Interactions between Buffer Ions and MC3

To provide evidence of our hypothesis of specific ion adsorption at the lipid/water interface, we performed all-atom MD simulations in explicit water. To ensure the correct protonation degree of the ionizable MC3 lipid, we chose an MC3/POPC monolayer system (Figure 5A) for which the area per lipid and protonation degree at pH 5.0 and 7.5 were determined consistently in previous work.^45^ In the simulations, citrate buffer was represented by the trivalent citrate ion, phosphate buffer by HPO_4_^2–^, and acetate buffer by the monovalent acetate ion. The affinity of the ions in terms of the probability distribution perpendicular to the lipid/water interface at pH 5.0 and 7.5 is shown in Figure 5B,C. The main adsorption peak at *z* = 0 nm corresponds to the interactions of the ions with the nitrogen moiety and follows a conventional Hofmeister ordering: citrate > phosphate > acetate. A second minor adsorption peak at *z* = −2 nm appears for acetate and corresponds to the interaction with the oxygen moiety. This peak is absent for the other ions and significantly smaller compared to the main adsorption peak. Further insights into the adsorption behavior at the interface can be gained from the local radial ion distributions *g*(*r*) around the charged and uncharged MC3 molecules. Figure 5D,E shows *g*(*r*) for the buffer ions around the N and O moieties (O1 representing the carbonyl O-moiety and O2 representing the ester O-moiety). For neutral MC3, the distributions at N- and O-moieties are similar (Figure 5D).

For charged MC3 (Figure 5E), pronounced differences between the ions and the different binding sites can be observed. The results show that citrate ions have a higher affinity toward the N atom in positively charged MC3 compared with the other buffer ions (as indicated by the highest peak in *g*(*r*) at a radial distance *r* = 0.28 nm in Figure 5E, top). Phosphate ions have the second highest affinity for the N-moiety followed by acetate. In the case of O-moieties, only acetate ions adsorb, while citrate and phosphate are depleted (Figure 5E, bottom). The simulation snapshots reveal that citrate ions are located at the lipid/water interface, while the acetate ions penetrate deeper into the lipid phase (Figure 5F,G). In summary, the affinity of buffer ions toward a lipid layer containing MC3 follows the Hofmeister series: citrate > phosphate > acetate. However, the local distribution of the ions is much more complex. The adsorption of acetate and depletion of phosphate and citrate at the oxygen is likely the cause of ion-specific buffer effects.

## Conclusions

In this work, mRNA-LNPs were prepared in three buffers, citrate, phosphate, and acetate. Transfection efficiency showed a dependence on the preparation buffer employed in the order citrate > phosphate > acetate for all three ionizable lipids investigated. To rationalize the buffer-specific efficiency, we recall that the core phase of LNPs exhibits a densely ordered lipid phase, which is predominantly formed by cationic ionizable lipid MC3 together with cholesterol and hence is pH-responsive. Note that DSPC and PEG-lipid are unlikely to partition into inverse phases. To explore the effect of buffer on pH response, we studied ionizable lipid-cholesterol bulk mesophases as core phase mimicking systems using SAXS synchrotron measurements. We find that the critical phase transition from inverse micellar to inverse hexagonal, specifically *Fd*3*m*-H_II_ in the case of MC3 and L_II_ -H_II_ in the case of SM-102 and ALC-315, is dependent on the buffer in the order citrate > phosphate ∼ acetate. In the case of MC3 lipid, the pH value of the transition shifts by 1 pH unit in citrate (6.5–5.5) compared to acetate (5.5–4.5). In earlier work, it was hypothesized that an analog structural transition occurs in the excess lipid regions inside LNPs and plays a critical role in the pH-dependent endosomal fusion process.^8,14^ The hypothesis that the structural transition of the LNP core induces endosomal fusion is plausible as the inverse micellar-to-inverse hexagonal transition causes defects destabilizing the surface monolayer of LNPs. A second mechanism associated with the pH-dependent structural transition involves the accumulation of the protonated ionizable lipid in the surface layer of LNPs at low pH. The resulting cationic surface charge consequently enhances the likelihood of endosomal fusion. A central question in our study is whether a theoretical explanation exists for the shift in the pH value of the *Fd*3*m*-H_II_ transition as a function of the specific buffer used. We propose that competition of elastic bending energy and charging of the ionizable lipid headgroup leads to the predicted shift. A surprising insight was that the protonated MC3 lipid exhibits a smaller headgroup area due to a conformational change in the headgroup, as seen by MD simulations. The dependence of the phase transition on the buffer is explained by specific ion adsorption at the nitrogen (N) and oxygen (O) moieties of the ionizable lipid, with a consequent change in the area per headgroup. This view is consistent with the finding that increasing the ionic strength stabilizes the H_II_ phase, likely favoring an even smaller headgroup area. By contrast, increasing the temperature to 37°C stabilizes the *Fd*3*m* phase due to a decrease in the p*K*_a_ of MC3, which favors the neutral lipid species, pushing the phase transitions toward lower pH. There is scope for further work to confirm our interpretation that the area per headgroup varies with buffer ion adsorption. It seems likely that acetate adsorption at the O-moiety, with an associated increase in area per headgroup, is responsible for the swelling trend observed in the H_II_ phase with the nearest neighbor distance (*d*_NN_) increasing with pH, while decreasing in the case of citrate and phosphate buffers.

The proposed mechanism presents a consistent structure–activity relation. The key assumption is that a pH-dependent structural transition in the excess lipid regions of the LNP core phase leads to endosomal fusion. For the argument to be conclusive, we must assume that the buffer ions used in the preparation of LNPs remain inside the LNPs and are not diluting out. A correlation of the transfection efficiency and bulk phase transition has been demonstrated here for three ionizable lipids: MC3, SM-102, and ALC-315. To what extent the structure–activity relation can be extended to other ionizable lipids remains to be determined. In recent work, it has been shown that the two mainstream COVID-19 vaccines ionizable ALC-315 and SM-102 lipids, show the same order of core phase changes as presented here.^8^ The proposed mechanism involves a direct buffer effect via specific adsorption of buffers but also an indirect effect via changes in the area per lipid headgroup consequent to buffer adsorption. In the case of MC3, a crucial finding is the role of buffer (acetate) adsorption at the O-moiety of the headgroup, distinct from interactions with the N-moiety carrying the headgroup charge. This suggests an avenue for engineering LNP behavior, manipulating the transition pH by functionalization of the headgroup beyond simply its ionizable character. Consistent with the notion of the lipid packing parameter,^11,39^ controlling the area per headgroup, and thereby the strength of the lipid layer bending energy, is key to controlling the transition. To achieve rational design strategies to exploit the described buffer-specific effects, theoretical models are required that explain the specific ion adsorption mechanism using MD simulation as well as an elastic continuum description of the mesophases. The consistent buffer-specific effects on LNP behavior demonstrate that understanding ionizable lipid mesophase transitions is useful for rationalizing mRNA transfection efficiencies and for further advancement of lipid formulations for gene therapy.

## Methods

### Materials

DLin-MC3-DMA(*O*-(*Z*,*Z*,*Z*,*Z*-heptatriaconta-6,9,26,29-tetraem-19-yl)-4-(*N*,*N*-dimethylamino) butanoate; (MC3, 99%), ALC-315 ([(4-hydroxybutyl)azanediyl]di(hexane-6,1-diyl) bis(2-hexyldecanoate)), and SM-102 (9-Heptadecanyl 8-{(2-hydroxyethyl)[6-oxo-6-(undecyloxy)hexyl]amino}octanoate) were purchased by MedChemExpress. 1,2-distearoyl-*sn*-glycero-3-phosphocholine, (DSPC, 99%), 1,2-distearoyl-*sn*-glycero-3-phosphoethanolamine-*N*-[amino(polyethylene glycol) (DSPE-PEG2000, 99%), cholesterol, sodium citrate dihydrate (99%), citric acid (99%), monobasic sodium phosphate (99%), disodium hydrogen phosphate (99%), hydrochloric acid (37%), sodium hydroxide (97%), sodium acetate (99%), acetic acid (99.8%), and citric acid (99.5%) were purchased by Avanti Polar lipid Sigma-Aldrich.

### Preparation of eGFP-mRNA LNPs

ARCA eGFP (Enhanced Green Fluorescent Protein) mRNA (APExBIO) was encapsulated in an LNP of four lipid components (DLin-MC3-DMA, DSPC, Cholesterol, and DMPE-PEG2000) in a 50:10:38.5:1.5 molar ratio. mRNA and buffer (pH 3) were prepared in aqueous solution (0.075 mg/mL) while the lipid components (1.11 mg/mL MC3, 0.27 mg/mL DSPC, 0.52 mg/mL cholesterol, and 0.15 mg/mL DSPE-PEG2k) were dissolved in ethanol. Concentrations were chosen to reach a final LNP concentration of 0.05 mg/mL mRNA concentration with an N/P ratio of 4 and a final buffer concentration of 50 mM. Microfluidic mixing was carried out with the NanoAssemblr Spark (Precision NanoSystems) at a volume ratio of aqueous:organic 2:1 ratio. Following mixing, the LNPs were incubated for 20 min at room temperature. To remove residual ethanol and buffers from the solution, LNPs were transferred to Slide-A-Lyzer MINI dialysis cups with 3.5 kDa molecular weight cutoff (ThermoFisher Scientific) and dialyzed into water for 18 h at room temperature. Size distribution was measured using a DynaPro NanoStar (Wyatt) DLS device. Encapsulation efficiency was assessed using the Quant-it RiboGreen RNA dye (Invitrogen).

### LNP Transfection Efficiency

Cells were cultured in RPMI 1640 Medium (ThermoFisher Scientific) supplemented with 10% (v/v) FBS (fetal bovine serum, ThermoFisher Scientific, no. 10270106), 5 mM HEPES (GibcoTM, Thermofisher Scientific, #15630080), and 1 mM Na-Pyruvate (GibcoTM, Thermofisher Scientific, #11360070) at 37°C, 5% CO_2_. For live-cell imaging, microstructures were prepared to allow single-cell culture. Therefore, six-channel μ-slides (ibidi) coated with cell-repellent PVA were treated with PLPP (*N*-(4-[benzoyl]benzyl)-*N*,*N*,*N*-triethylammonium bromide) (enamine) in an agarose and calcium peroxide solution and selectively illuminated with UV light (365 nm). Selective illumination was facilitated with a photomask patterned with 20 × 20 μm squares and an 80 μm spacer. After illumination, the channels were rinsed with water and 0.5 M HCl. The resulting squares were coated with laminin by incubation in a 20 μg/mL laminin (BioLamina) working solution in PBS for 1 h at 37°C.

Application of cells in the medium for 1 h led to self-assembly in a single cell pattern, as depicted in Figure 2A. eGFP-mRNA-LNPs were diluted in RPMI medium supplemented with FCS to a final concentration of 1 ng/μL and incubated for 1 h at room temperature to allow the formation of a protein corona. Subsequently, the LNP solution was applied for 1 h and washed afterward with L15-medium without phenol red (ThermoFisher Scientific). Cells were transferred to the microscope (Nikon TI Eclipse) and imaged over 30 h with image acquisition every 10 min. Image analysis was then performed using our in-house Python-based software including segmentation and background correction based on Schwarzfischer et al.^46^ to generate fluorescence trajectories.

### Bulk Phase Preparation: Dialysis Steps

MC3/cholesterol bulk phases were prepared under a range of pH conditions via three dialysis steps. First, the MC3 and cholesterol were dissolved in ethanol and mixed in a molar ratio of 3:1 (MC3: cholesterol) to a total lipid concentration of 56.1 mg/mL (MC3 46.7 mg/mL and cholesterol 9.4 mg/mL). The mixture was put into a dialysis cup with a molecular weight cut off of 3.5 kDa. Samples were first dialyzed against a 50 mM citrate buffer (pH 3) containing ethanol (at a volume ratio of 3:1, buffer: ethanol) for 48 h. In the second dialysis step, the sample was dialyzed against PBS (1 mM KH_2_PO_4_, 155 mM NaCl, 3 mM Na_2_HPO_4_ 0.7 H_2_O, pH 7.4) for 48 h. In the third step, the sample was dialyzed against NaCl 150 mM and the buffer of choice (citrate, acetate, or phosphate 50 mM at pH 3.5, 4.0, 4.5, 5.0, 5.5, 6.0, 6.5, 7.0) with the required final pH for 48 h. The samples at different ionic strengths (*I*) were prepared at different acetate, phosphate, and citrate concentrations to reach *I* = 160 mM, *I* = 200 mM, and *I* = 300 mM including the background salt NaCl 150 mM. The supernatant was removed from the cup and the solid precipitates were extracted for characterization by SAXS.

### SAXS Measurements

Synchrotron small-angle X-ray scattering (SAXS) was carried out at the P12 EMBL BioSAXS and P62 SAXSMAT beamlines, PETRA III, DESY (Hamburg, Germany). The beamline instrumentation has been described previously.^47,48^ Further SAXS experiments were performed using an internal instrument at LMU. The crystallographic space groups of the liquid crystalline phases were determined from the relative peak positions. All of the measurements at P12 and P62 were performed in quartz capillaries. The scattering data background was subtracted by measuring the empty capillaries.

### Calculation of Nearest Neighbor Distance *d*_NN_

The nearest neighbor distance was calculated for each mesophase using the peak positions with their respective Miller indices following the formula shown in Tables S2 and S3 (see the Supporting Information).^14^

### All-Atom Molecular Dynamics Simulations

Ion-specific adsorption simulation setup: To investigate the adsorption of acetate, phosphate, and citrate at the lipid/water interface, we used a monolayer setup. The lipid monolayer contained MC3 and POPC lipids in a 1:4 molar ratio and was constructed using the Men-Gen web server^49^ following the published procedure of our previous work.^45^ Each leaflet of the monolayer contained 200 lipids separated by a water column of approximately 15 nm. The reason for choosing this setup was that the protonation degree at two pH values was determined consistently from a combination of simulations and scattering experiments.^45^ This was crucial since the degree of protonation is inherently difficult to predict since it depends on the local lipid environment and is not directly accessible from experiments. Specifically, the lipid monolayer at pH 5 has a protonation degree of 67.5% ionizable MC3. At pH 7.5, the protonation degree is 14.5%. Both values significantly deviate from simple theoretical predictions. For each pH value, the monolayers were simulated at a buffer concentration of 50 mM. Sodium acetate (CH_3_COONa), sodium phosphate (Na_2_HPO_4_), and sodium citrate (Na_3_C_6_H_5_O_7_) were used in the experiments. Thus, a total of six simulations of the lipid monolayer were performed, covering two pH levels and three different buffers. Each simulation was repeated three times to improve the sampling statics.

The AMBER Lipid 17 force field was used to describe the POPC lipids. For the cationic and neutral MC3 molecules, we used recently developed force fields, which closely reproduce the experimental structure of lipid layers and are compatible with the AMBER force field family.^50^ The TIP3P water model^51^ along with Mamatkulov–Schwierz^52^ force field parameters for Na^+^ ions was also used. The citrate ion was described using the force field parameters obtained from Wright^53^ while the acetate and phosphate ion force field parameters were obtained from Kashefolgheta.^54^ The combination rule for the anion–cation interactions was modified to avoid crystallization artifacts. The resulting force field parameters are available at “(https://git.rz.uni-augsburg.de/cbio-gitpub/force-fields-buffer-ions).”

All-atom molecular dynamic simulations were performed using the GROMACS^55^ package (v-2024). A gradient descent algorithm was used to minimize the energy of the system. The simulations were performed in the *NVT* ensemble to ensure the correct area per lipid.^50^ The temperature was maintained at 293.15 K using the velocity rescale thermostat with a time constant of 1.0 ps. The Lennard–Jones potential was cut off and shifted to zero at 1.2 nm. Short-ranged electrostatic interactions were cutoff at 1.2 nm, and the Particle Mesh Ewald (PME) method was used to evaluate long-range electrostatics. Hydrogen bonds were constrained using the LINCS algorithm, and a time step of 2 fs was used. Each production run was performed for 70 ns. The first 20 ns of the simulation were discarded to account for equilibration and the rest of the trajectory was analyzed using GROMACS inbuilt modules and MDAnalysis.^56^ The trajectories were visualized, and snapshots were generated using visual molecular dynamics (VMD).^57^

Area per headgroup of H_II_ and L_II_ phases: to obtain the area per headgroup, we used data from our previous work,^14^ where we simulated the inverse hexagonal (H_II_) and inverse micellar (L_II_) phases. The H_II_ phase consisted of fully protonated DLin-MC3-DMA (MC3) lipids combined with cholesterol in a 3:1 molar ratio, maintaining a water-to-lipid ratio (*n*_w_) of 12 to match the experimental lattice spacing of 60 Å. The L_II_ phase consisted of fully uncharged MC3 lipids using also *n*_w_ = 12. Details on the calculation of the area per headgroup are provided in the Supporting Information.

Content submitted to preprint server bioRxiv, Authors: Cristina Carucci, Julian Philipp, Judith A. Müller Akhil Sudarsan, Ekaterina Kostyurina, Clement E. Blanchet, Nadine Schwierz, Drew F. Parsons, Andrea Salis, Joachim O. Rädler. Title: Buffer specificity of ionizable lipid nanoparticle transfection efficiency and bulk phase transition. 2025, DOI: 10.1101/2025.01.17.63350. Repository: bioRxiv, the preprint service for biology. 10.1101/2025.01.17.633509 (version accessed January 21, 2025).

## Supporting Information Available

SAXS measurements of the ionic strength and temperature effects in the presence of buffer citrate and acetate, DLS data of LNPs, Miller indices of the measured lipid phases together with formula of the lattice parameter, SAXS measurements of mesophase of MC3-cholesterol, ALC-315-cholesterol, SM-102-cholesterol samples with Miller indices in buffer citrate, phosphate, and acetate, *d*_NN_ values for H_II_ and L_II_ phases for ALC-315-cholesterol and SM-102- cholesterol samples as a function of pH for buffer citrate, phosphate, and acetate, molecular simulations methods including adjustment of combination rules for the force field parameters, force field parameters, and calculation of area per lipid, and London dispersion coefficients of buffer ions in water and in nonpolar media (PDF)
