# Supplementary material for: Buffer Specificity of Ionizable Lipid Nanoparticle Transfection Efficiency and Bulk Phase Transition
Source: ACS Nano. 2025 Mar 12;19(11):10829–40. doi: 10.1021/acsnano.4c14098 (PMC11949115; doi:10.1021/acsnano.4c14098)
Supplement: Supplementary file 1 — nn4c14098_si_001.pdf [file nn4c14098_si_001.pdf]

**Supporting Information**

# Buffer specificity of ionizable lipid nanoparticle transfection efficiency and bulk phase transition

*Cristina Carucci[1,‡], Julian Philipp[2,‡], Judith A. Müller[2], Akhil Sudarsan[3], Ekaterina Kostyurina[2], Clement E. Blanchet[4], Nadine Schwierz[3], Drew F. Parsons[1,*], Andrea Salis[1], Joachim O. Rädler[2,*]*

[1]Department of Chemical and Geological Sciences, University of Cagliari & Center for Colloid and Surface Science (CSGI),
Cittadella Universitaria, 09042 Monserrato, CA, Italy

[2]Faculty of Physics, Ludwig-Maximilians University,
Geschwister-Scholl-Platz 1, Munich, Germany.

[3]Institute of Physics, University of Augsburg, Augsburg 86159, Germany.

[4]European Molecular Biology Laboratory Hamburg Outstation c/o Deutsches Elektronen-Synchrotron, Hamburg 22607, Germany.

‡ CC and ‡ JP contributed equally to the work.

*Prof. Joachim Radler, raedler@lmu.de

*Prof. Drew Parsons, drew.parsons@unica.it

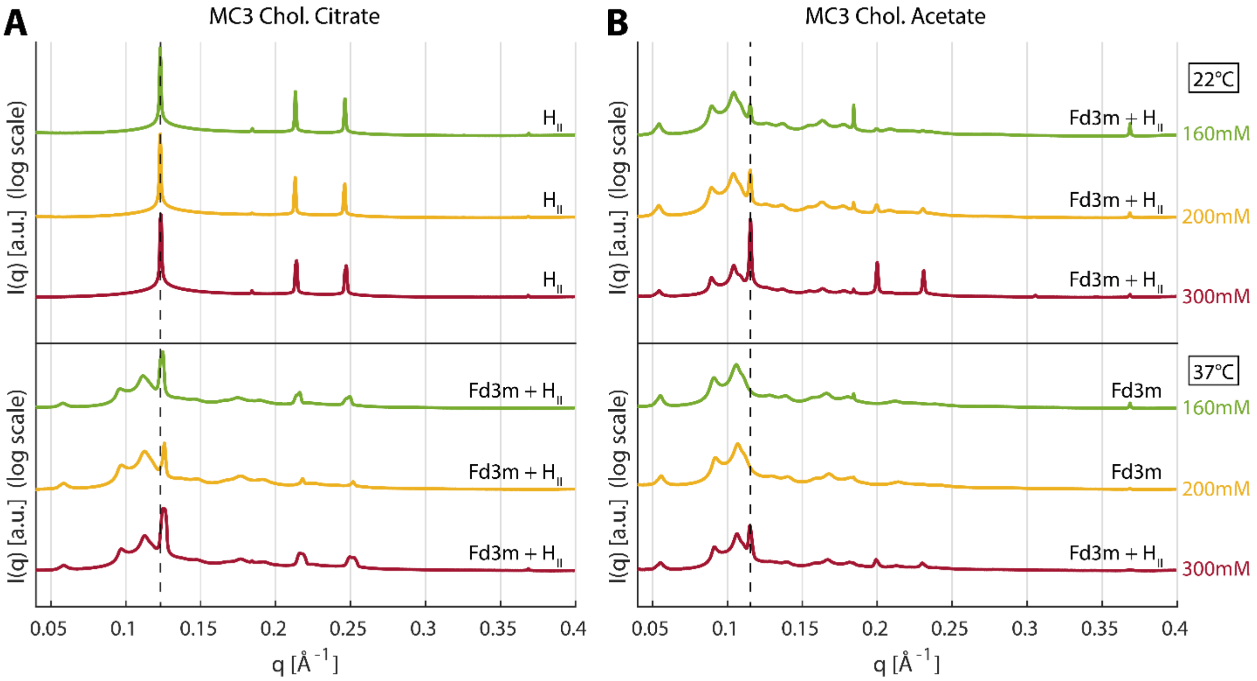


**Figure S1.** SAXS measurements of MC3-cholesterol dialyzed in the presence of 160-, 200-and 300-mM ionic strength citrate (A) and acetate (B) buffer at 22 °C and 37 °C at pH 5.5. Measurements of ionic strength (*I*) include the presence of NaCl 150 mM as background salt.

**Table S1.** Hydrodynamic diameter $d_H$, polydispersity index (PDI) as indication of the size measurements quality associated to the size distribution and zeta potential measurements of MC3 LNPs prepared in acetate 155 mM, phosphate 100 mM, and citrate 50 mM buffers at pH 4.5.

| **Buffer** | **$d_H$ (nm)** | **PdI** | **pH*** |
|---|---|---|---|
| **Citrate** | 76 ± 7 | 0.330 | 5.75 ± 0.25 |
| **Phosphate** | 70 ± 1 | 0.304 | 5.3 ± 0.5 |
| **Acetate** | 73 ± 2 | 0.324 | 5.25 ± 0.25 |

*pH of Fd3m-$H_{II}$ transition

**Table S2.** Miller indices of the measured lipid phases. $a$ is the lattice parameter and $d_{hkl}$ the corresponding real-world distance. This table is taken from reference [1].

| **Lipid phase** | **Miller indices** $(hkl)$ | $a/d_{hkl}$ | $m_{hkl}$ |
|---|---|---|---|
| $H_{II}$ | $(10), (11), (20), (21), (30), \ldots$ | $\sqrt{h^2+k^2+hk}$ | $1, \sqrt{3}, 2, \sqrt{7}, \sqrt{9}, \ldots$ |
| $Fd3m$ | $(111), (220), (311), (222), (400),$ | $\sqrt{h^2+k^2+l^2}$ | $\sqrt{3}, \sqrt{8}, \sqrt{11}, \sqrt{12}, 4, ..$ |
| $P\,6_3/mmc$ | $(010), (002), (011), (012), (110),$ | $\frac{4}{3}(h^2+k^2+hk)+\frac{3}{8}l^2$ | $\sqrt{\frac{4}{3}}, \sqrt{\frac{3}{2}}, \sqrt{\frac{41}{24}}, \sqrt{\frac{17}{6}}, 4$ |

**Table S3.** Formula of the lattice constant $a$ and the nearest neighbor distance $d_{NN}$ for the measured lipid phases using the peak positions $q_{hkl}$ as well as $m_{hkl}$ from Table 2. This table is taken from reference [1].

| **Lipid phase** | $a$ | $d_{NN}$ |
|---|---|---|
| $H_{II}$ | $\frac{4\pi m_{hk}}{\sqrt{3}q_{hk}}$ | $a$ |
| $Fd3m$ | $\frac{2\pi m_{hkl}}{q_{hkl}}$ | $\frac{a}{\sqrt{8}}$ |
| $P6_3/mmc$ | $\frac{2\pi m_{hkl}}{q_{hkl}}$ | $a$ |

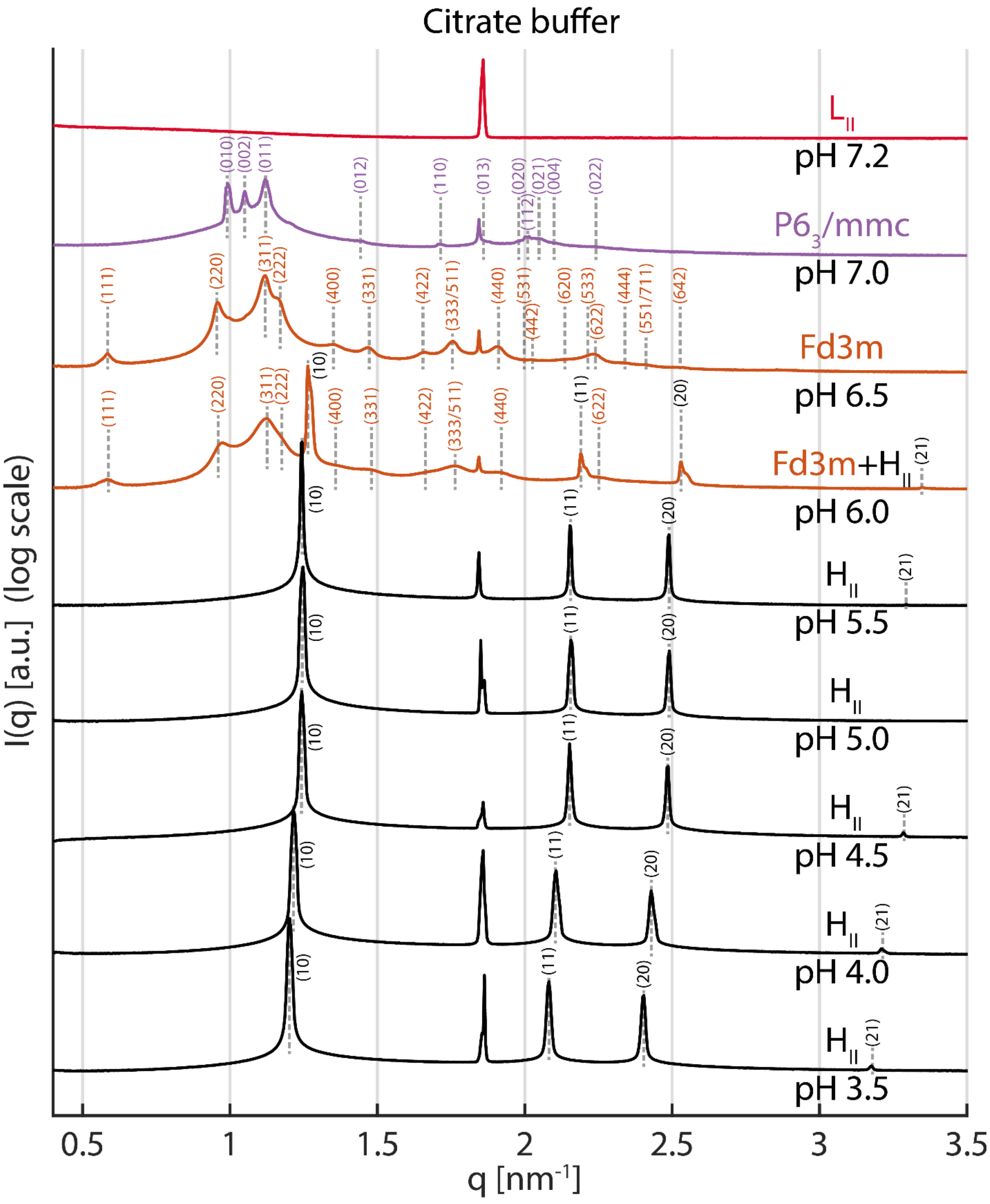


**Figure S2.** SAXS measurements and Miller indices of the mesophase of MC3-cholesterol samples dialyzed in the presence of 50 mM citrate buffer and NaCl 150 mM in a range of pH 3.5 - 7.2.

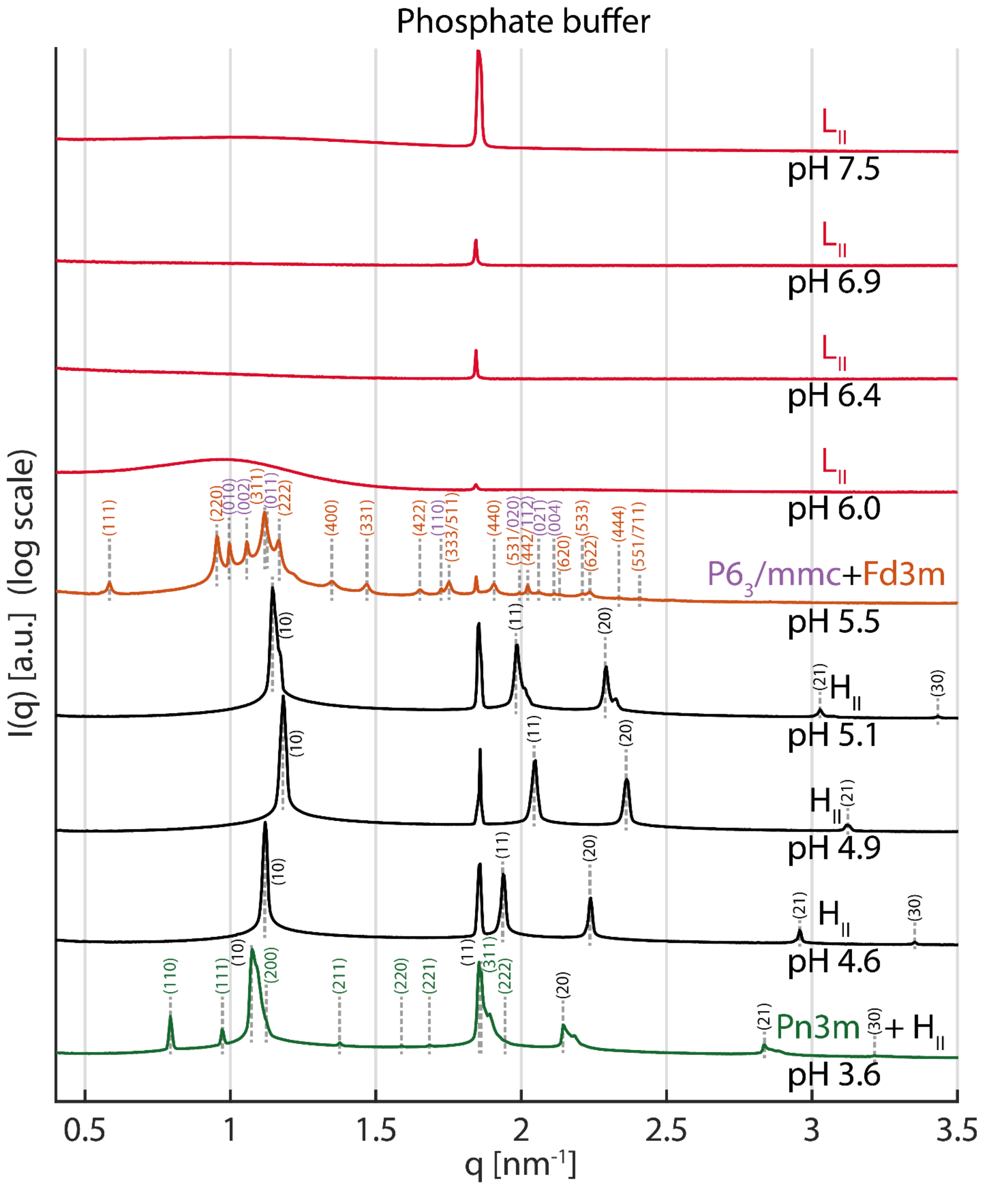


**Figure S3.** SAXS measurements and Miller indices of the mesophase of MC3-cholesterol samples dialyzed in the presence of 50 mM phosphate buffer and NaCl 150 mM in a range of pH 3.6 - 7.5.

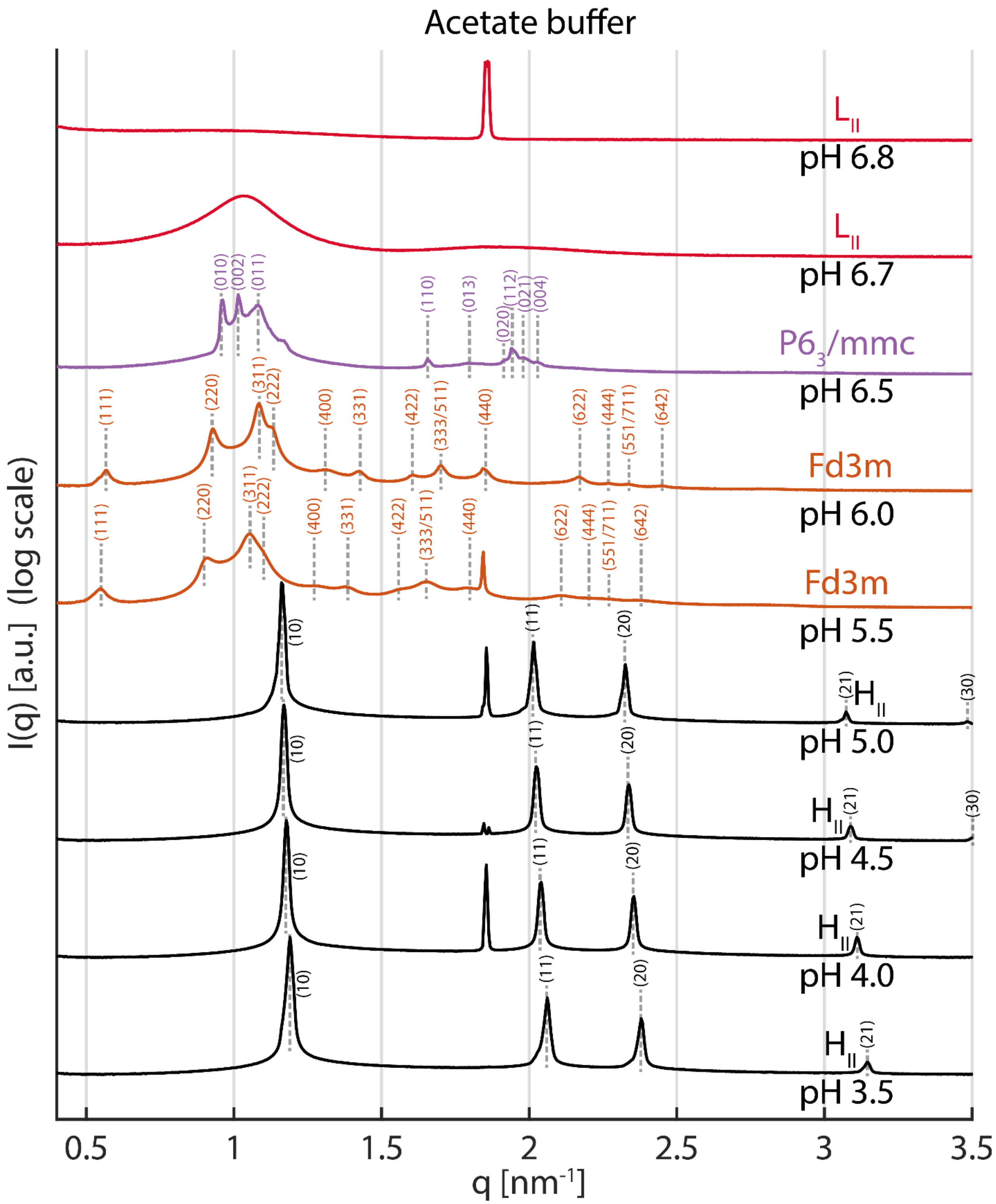


**Figure S4.** SAXS measurements and Miller indices of the mesophase of MC3-cholesterol samples dialyzed in the presence of 50 mM acetate buffer and NaCl 150 mM in a range of pH 3.5 - 6.8.

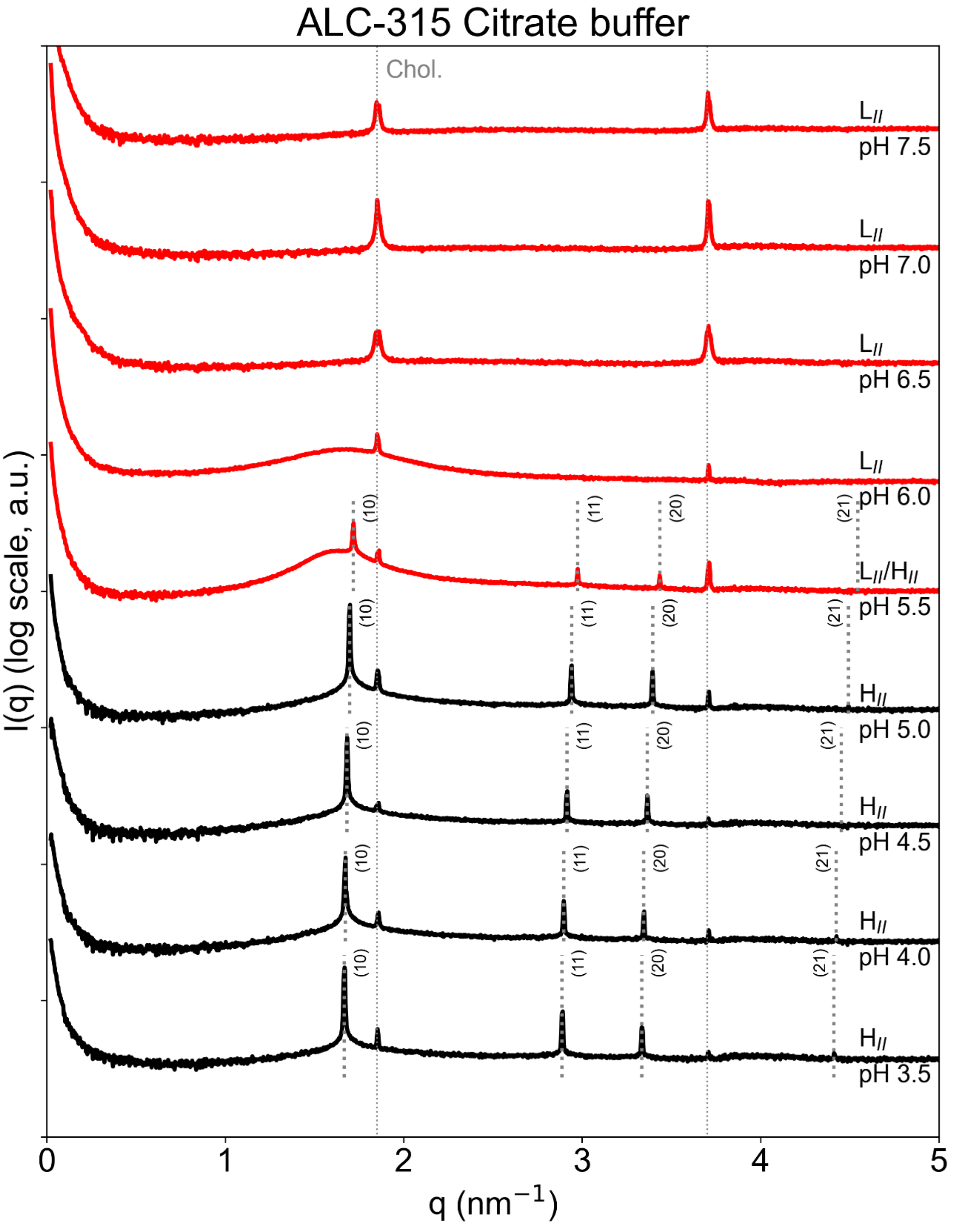


**Figure S5.** SAXS measurements of the mesophase of ALC315-cholesterol samples dialyzed in the presence of 50 mM citrate buffer and NaCl 150 mM in a range of pH 3.5- 7.5.

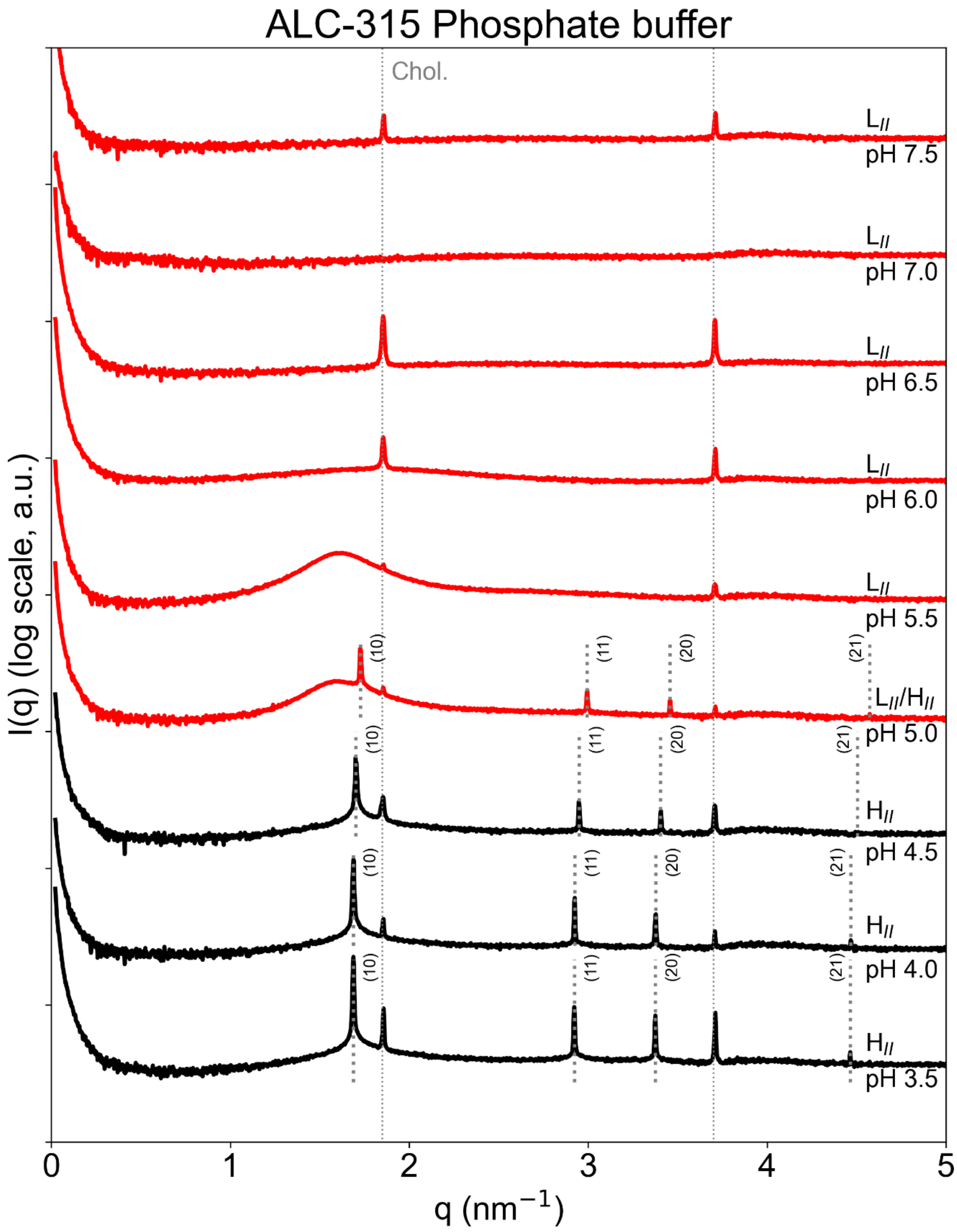


**Figure S6.** SAXS measurements of the mesophase of ALC315-cholesterol samples dialyzed in the presence of 50 mM phosphate buffer and NaCl 150 mM in a range of pH 3.5- 7.5.

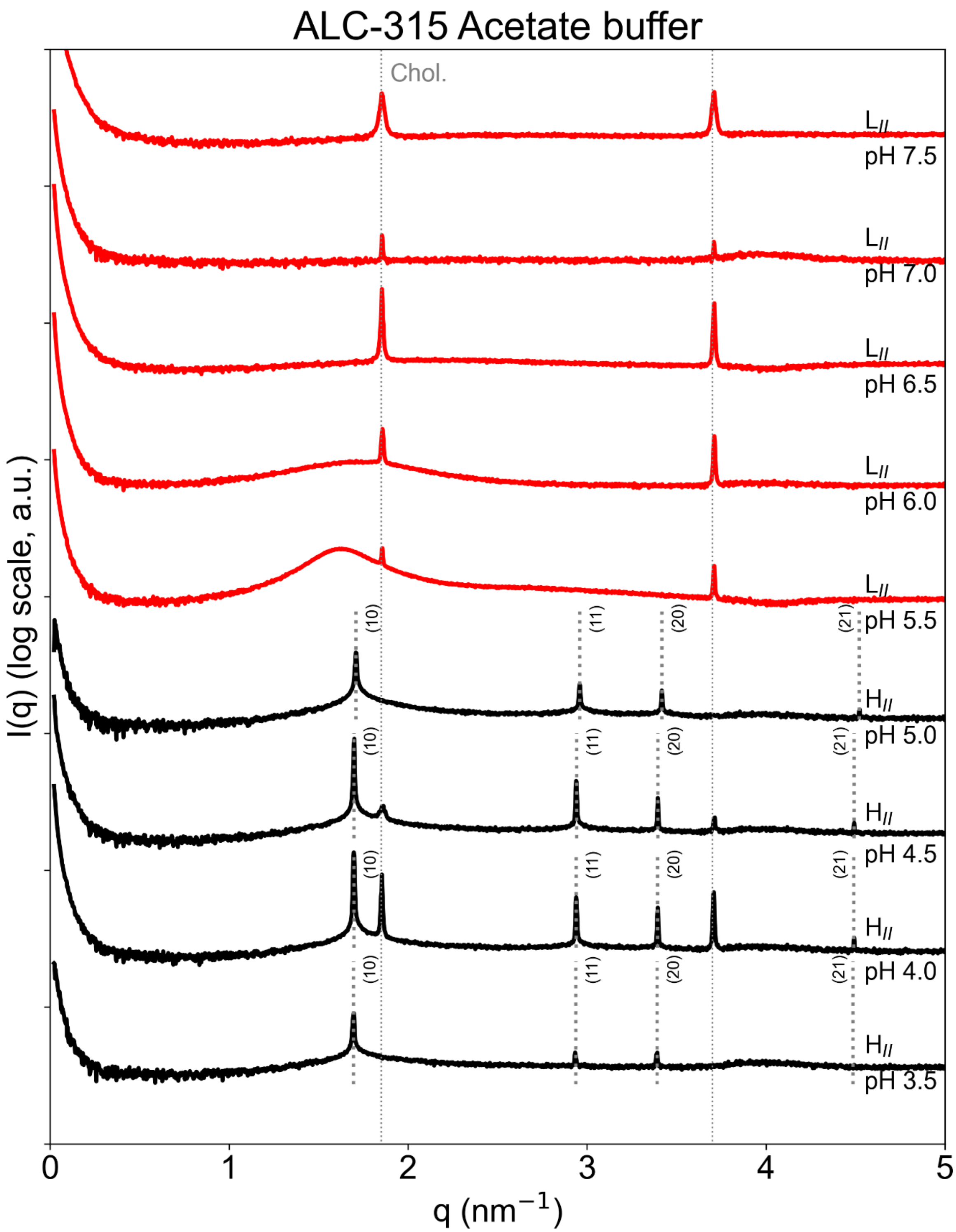


**Figure S7.** SAXS measurements of the mesophase of ALC315-cholesterol samples dialyzed in the presence of 50 mM acetate buffer and NaCl 150 mM in a range of pH 3.5- 7.5.

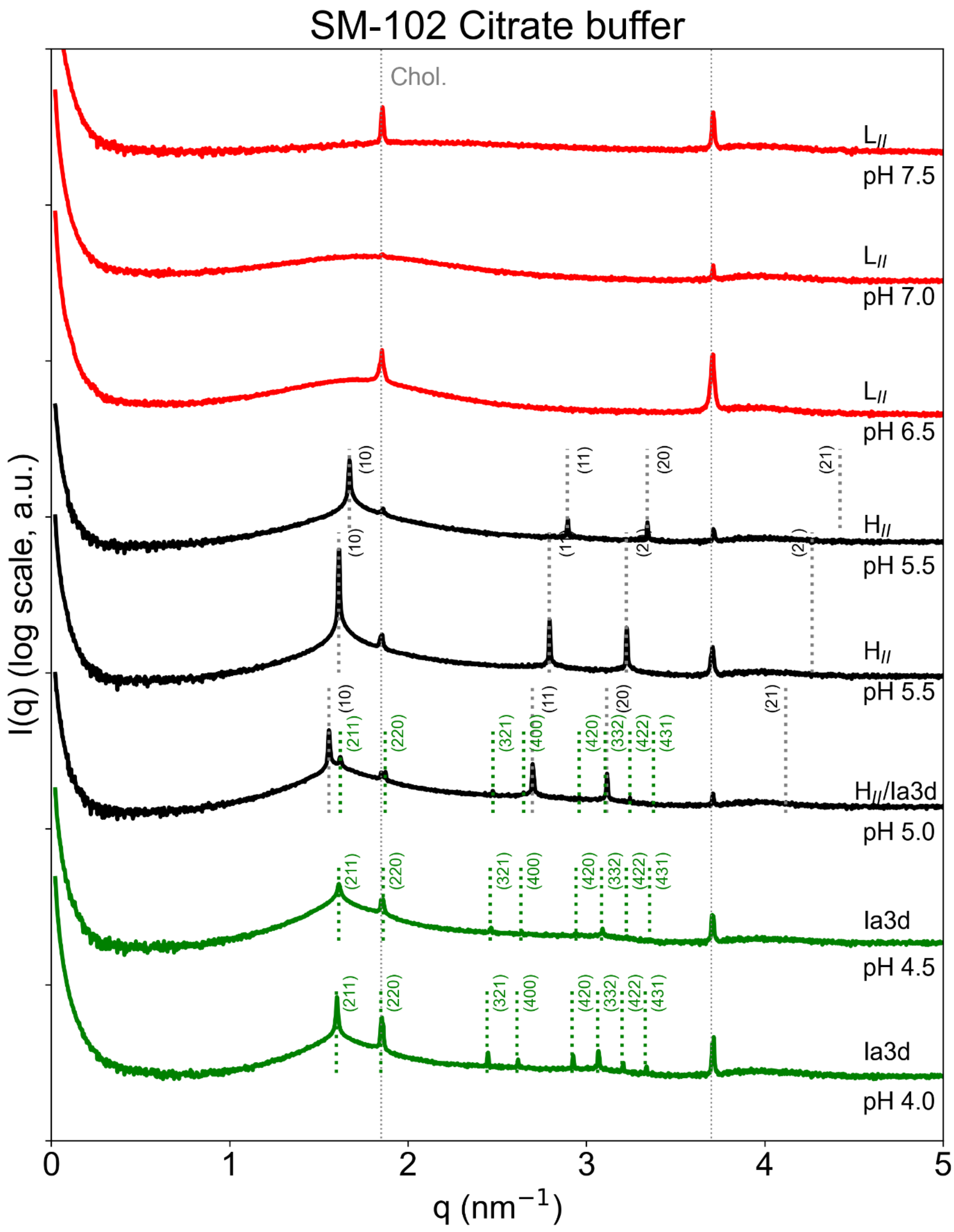


**Figure S8.** SAXS measurements of the mesophase of SM102-cholesterol samples dialyzed in the presence of 50 mM citrate buffer and NaCl 150 mM in a range of pH 4.0 - 7.5.

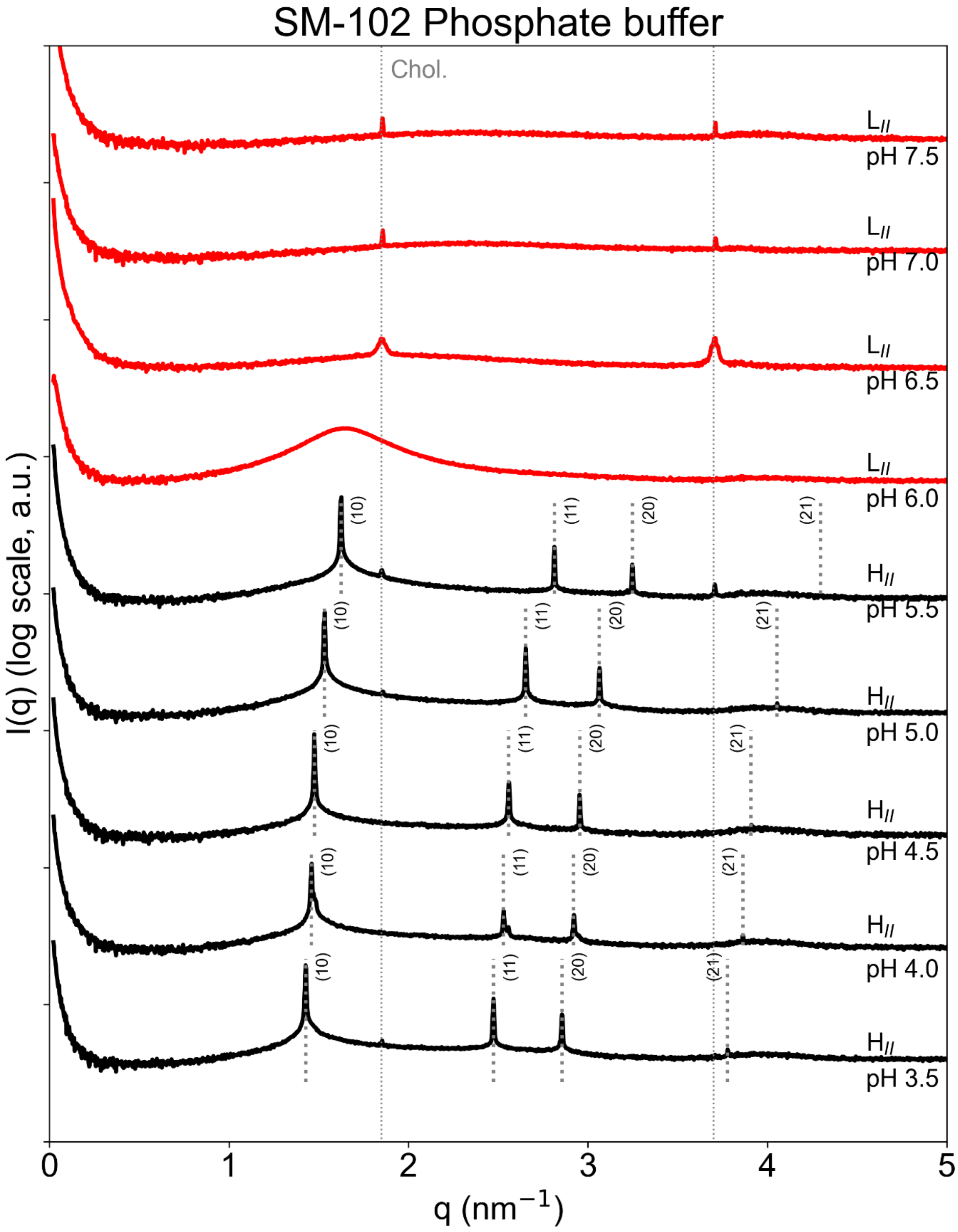


**Figure S9.** SAXS measurements of the mesophase of SM102-cholesterol samples dialyzed in the presence of 50 mM phosphate buffer and NaCl 150 mM in a range of pH 3.5 - 7.5.

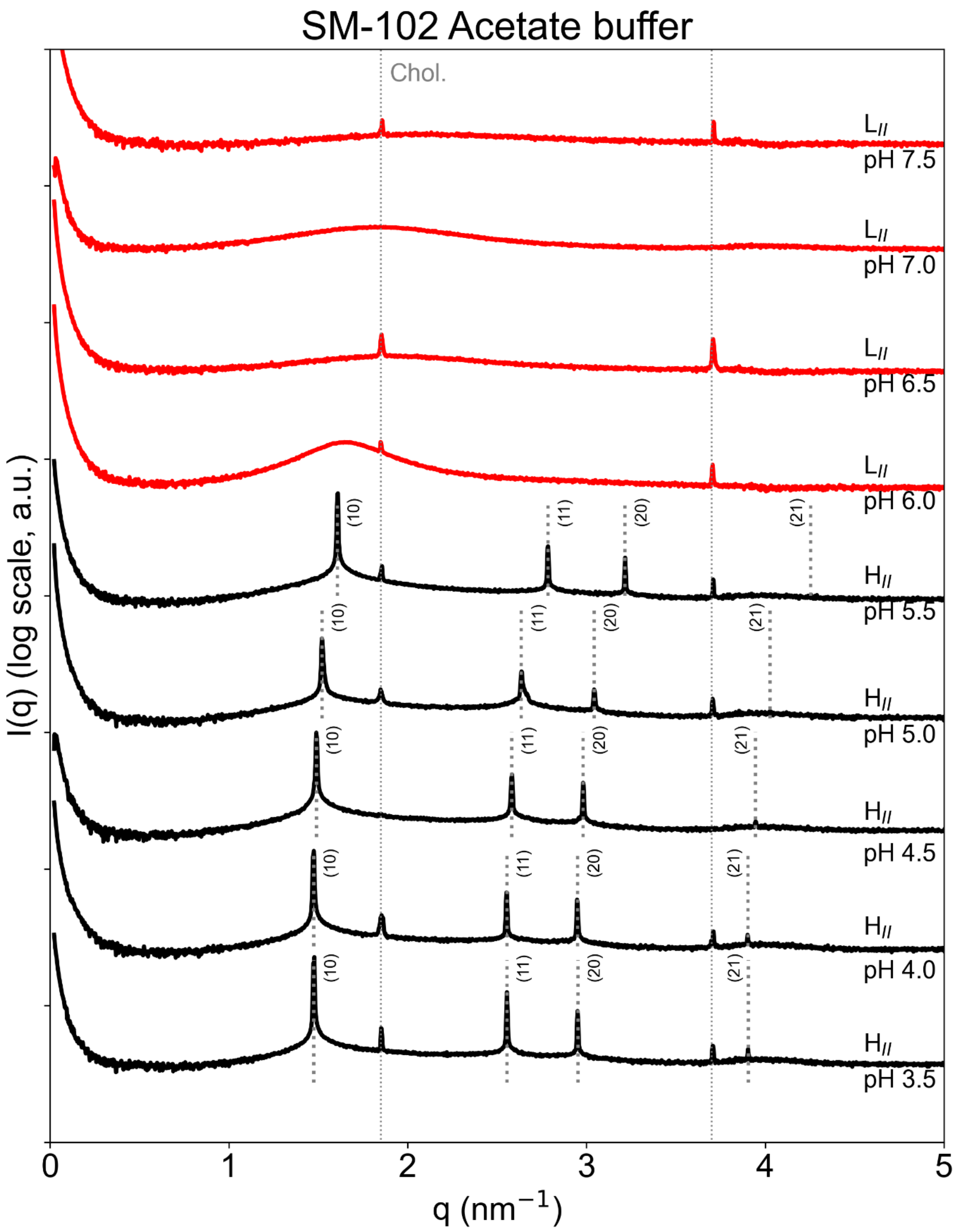


**Figure S10.** SAXS measurements of the mesophase of SM102-cholesterol samples dialyzed in the presence of 50 mM acetate buffer and NaCl 150 mM in a range of pH 3.5 - 7.5.

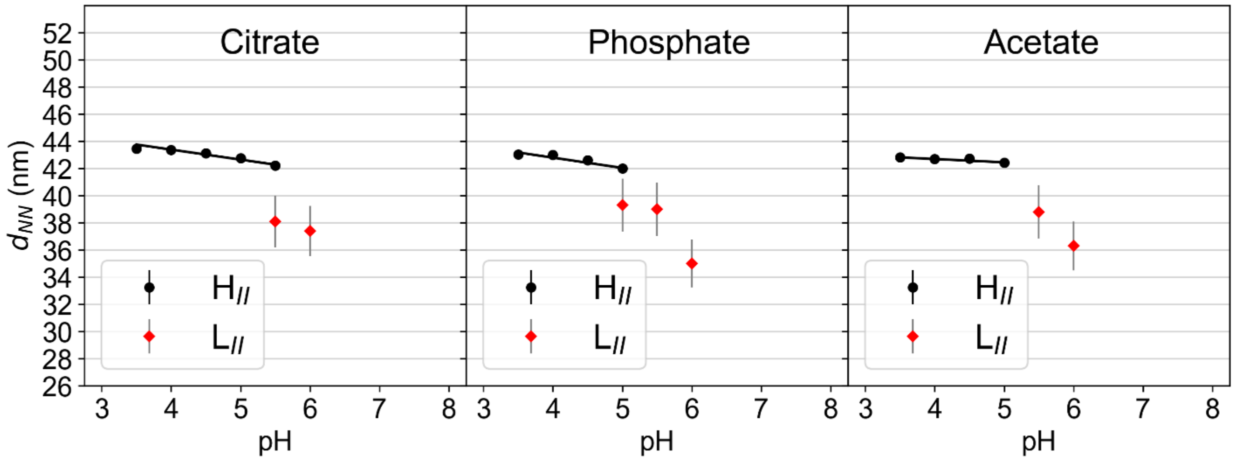


**Figure S11**. $d_{NN}$ values for $H_{II}$ and $L_{II}$ phases for ALC315-cholesterol samples as a function of pH for buffer citrate, phosphate and acetate.

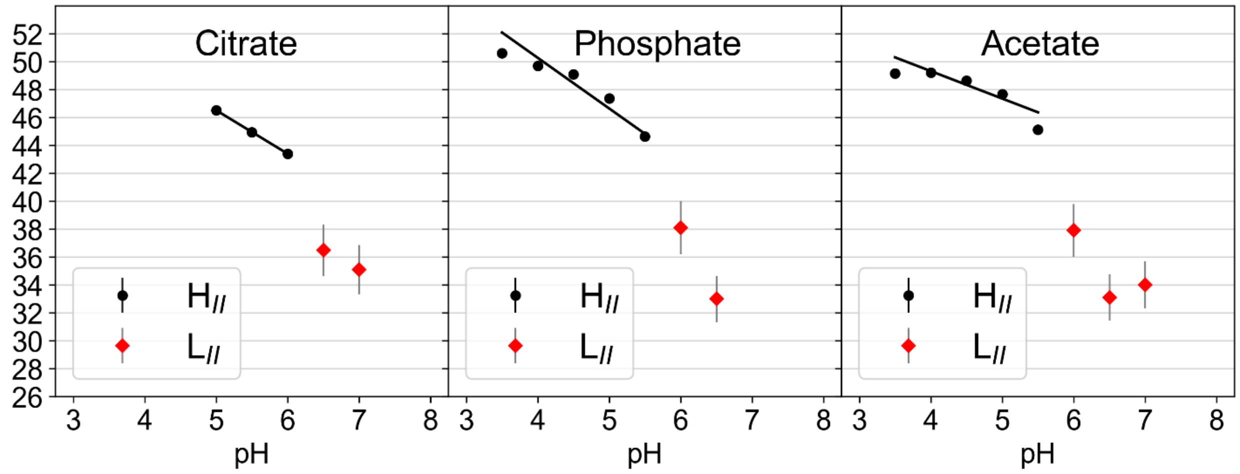


**Figure S12**. $d_{NN}$ values for $H_{II}$ and $L_{II}$ phases for SM102-cholesterol samples as a function of pH for citrate, phosphate and acetate.

## Molecular simulation methods

### Adjustment of combination rules for the force field parameters

In MD simulations, the non-bonded interaction potential of the ions has the following form:

$$V(r_{ij}) = \frac{q_i q_j}{4\pi\epsilon_o r_{ij}} + 4\epsilon_{ij}\left[\left(\frac{\sigma_{ij}}{r_{ij}}\right)^{12} - \left(\frac{\sigma_{ij}}{r_{ij}}\right)^{6}\right] \qquad (1)$$

The first term describes the electrostatic interactions via a Coulomb potential. The last two terms describe the Van-der-Waal interactions via a Lennard-Jones (LJ) interaction potential. $q_i$ and $q_j$ are the charges of atoms $i$ and $j$ respectively, $r_{ij}$ is the interatomic distance, $\sigma_{ij}$ describes the LJ diameter at which the potential is zero and $\epsilon_{ij}$accounts for the interaction strength. MD simulations of citrate and phosphate ions at finite concentrations resulted in crystallization of the ions. This effect was observed for different $Na^+$ force fields including the ones from ref. 50, 52 of the main manuscript. To avoid this artifact, we systematically increased the anion-cation LJ diameter by introducing a scaling factor in the corresponding Lorentz-Bertholot combination rule:

$$\sigma_{ij} = \lambda_{ij}\frac{\sigma_i + \sigma_j}{2} \qquad (2)$$

The value of $\lambda_{ij}$ was increased until no more clustering was observed as judged by the anion-cation radial distribution functions. The final values are $\lambda=2$ for the citrate-sodium scaling and the phosphate-sodium interaction. Only the interaction of the sodium ions with the oxygen atoms of the phosphate and citrate ions were scaled. Note that in a more rigorous approach, the experimental

activity derivative could be used to further optimize the anion-cation interactions as in previous work in reference 2.

**Force Field parameters for phosphate**

The Lennard-Jones force field parameters for the phosphate ions ($HPO_4^{2-}$) were taken from $H_2PO_4^-$ in ref 3. The partial charges were obtained from RESP fitting.

**Calculation of area per lipid**

The area per lipid of both the $L_{II}$ and $H_{II}$ phases were calculated for the system with $n_w = 12$. In the $H_{II}$ phase, which is enclosed within a triclinic unit cell characterized by a lattice spacing $d_H$ and height $h$, we assume that the water column is an equally long cylinder with a radius $R_H$. The water volume fraction (H) of the $H_{II}$ phase is obtained by dividing the volume of the water column by the total volume of the system. The total volume of the system in the $H_{II}$ phase is the volume of the triclinic unit cell which is given by $d_H^2 h$ sin(60°). The water volume fraction $\phi_H$ is given by:

$$\varphi_H = \frac{\pi R_H^2 h}{d_H^2 h\, Sin(60°)} \qquad (3)$$

From this equation, the radius $R_H$ of the cylindrical water column can be derived as

$$R_H = d_H \sqrt{\frac{\sqrt{3}}{2\pi}\phi_H} \qquad (4)$$

The value of H = 0.24 and $d_H$ = 60 Å is taken from ref. 1. The area per lipid *(A)* of the f the $H_{II}$ phase is obtained by dividing the surface area of the water column by the total number of lipids *N*.

$$A = \frac{2\pi R_H h}{N} \qquad (5)$$

Similarly, for the $L_{II}$ phase simulated in a rhombic dodecahedron box of lattice spacing *dL*, we assume a water sphere of radius $R_L$ surrounded by lipids. The water volume fraction (L) of the $L_{II}$ phase is obtained by dividing the volume of the water sphere by the total volume of the system. For a rhombic dodecahedron box of length *dL*, the volume of the box is 0.707 $d_L^3$ (i.e. $d^3/\sqrt{2}$). The water volume fraction $\phi_L$ is given by

$$\phi_L = \frac{(4/3)\pi R_L^3}{0.707\ d_L^3} \qquad (6)$$

The radius of the water sphere $R_L$ can be obtained from the above equation by:

$$R_L = 0.55\ d_L(\phi_L)^{\frac{1}{3}} \qquad (7)$$

Corresponding area per lipid of the $L_{II}$ phase,

$$A_L = \frac{4\pi {R_L}^2}{N} \qquad (8)$$

**Calculation of probability distributions**

The probability distributions for the ions perpendicular to the interface were calculated as

$$P(z) = \frac{\rho(z)}{\int_{-D/2}^{D/2} \rho(z)dz} \tag{9}$$

where $D$ is the distance between the monolayers and ρ(z) is the number density of the ions. The probability distributions are normalized such that $\int_{-D/2}^{D/2} \rho(z)dz = 1$.

**Calculation of radial distribution function**

The radial distribution function $g(r)$ provides insights into the local distribution of the ions around the protonated and neutral MC3 lipids. The radial distribution functions were obtained from the following equation:

$$g(r) = \frac{n(r)}{N_R \rho 4\pi r^2 dr} \tag{10}$$

n(r) is the count of buffer ions around the reference atoms at distance $r$, $N_R$ is the number of reference atoms, and $\rho = N_I/V_w$ is the bulk density of the ions. $N_I$ is the total number of anions in the system minus the anions used for neutralization and $V_w$ is the volume of water obtained by multiplying the theoretical molecular volume of a water molecule with the total number of water molecules in the system. In a bulk solution, $g(r)$ gives the number of ions at distance $r$ relative to the number of ions in an ideal solution. At an interface, such as the lipid water interface investigated here, equation (8) gives the number of buffer ions at distance r relative to a uniform ion distribution in the same slab geometry. The MD analysis python module (reference 4) was

used to count the number of anions around the reference atoms (N, O1 and O2) of the charged and uncharged MC3 lipid head group using the simulation setup at pH 5.

**London dispersion interactions and the O-moiety**

Acetate ion binding to the O-moiety of the MC3 headgroup can be understood in part from direct London dispersion interactions, $U(d) = -C/d^6$, where $d$ is the distance between the ion and the O-moiety. The London dispersion coefficient may be evaluated from quantum mechanical electronic polarizabilities of the species by applying quantum electrodynamics methods as reported in reference 5,6. Table S4 presents calculated London dispersion coefficients of buffer ions with a neutral acetic molecule representing the O-moiety (i.e. representing with O-moiety via a simple bound carbonyl group). London coefficients are evaluated in aqueous medium, and in nonpolar medium (tetradecane).

**Table S4.** London dispersion coefficients of buffer ions with acetic acid (representing the MC3 O-moiety) in aqueous media and in non-polar medium

| Buffer ion | *C*, aqueous (Å$^6$ kJ/mol) | *C*, in tetradecane |
|---|---|---|
| acetate | 5414 | 3752 |
| $H_2PO_4^-$ | 5635 | 4473 |
| $HPO_4^{2-}$ | 4188 | 1722 |
| $PO_4^{3-}$ | -2069 | -8693 |
| $H_2$-citrate$^-$ | 2182 | 149 |
| H-citrate$^{2-}$ | 1456 | -1347 |
| citrate$^{3-}$ | -258 | -4635 |

The London coefficient is significantly more attractive for acetate ion than any of the citrate species. Curiously, trivalent citrate even has a negative coefficient, indicating a repulsive

interaction pushing citrate away from the representative O-moiety. The aqueous London coefficient for phosphate is similar to acetate, indicating that this mechanism does not solely control the binding of acetate to the O-moiety observed in MD simulations. The O-moiety is located deeper inside the headgroup layer, such that the environment of the interaction is not purely aqueous. The reduced polar environment deep inside the head group is more unfavorable to phosphate than to acetate, which is surface active (partially oleophilic) due to its short hydrocarbon group. Indeed, the London coefficient of acetate in a nonpolar environment (tetradecane) is much stronger (more attractive) than all phosphate and citrate species apart from $H_2PO_4^-$.

## REFERENCES


(1) Philipp, J.; Dabkowska, A.; Reiser, A.; Frank, K.; Krzysztoń, R.; Brummer, C.; Nickel, B.; Blanchet, C. E.; Sudarsan, A.; Ibrahim, M.; Johansson, S.; Skantze, P.; Skantze, U.; Östman, S.; Johansson, M.; Henderson, N.; Elvevold, K.; Smedsrød, B.; Schwierz, N.; Lindfors, L.; Rädler, J. O. PH-Dependent Structural Transitions in Cationic Ionizable Lipid Mesophases Are Critical for Lipid Nanoparticle Function. *Proc. Natl. Acad. Sci.* **2023**, *120*, 2017.

(2) Fyta, M.; Netz, R. R. Ionic Force Field Optimization Based on Single-Ion and Ion-Pair Solvation Properties: Going beyond Standard Mixing Rules. *J. Chem. Phys.* **2012**, *136*.

(3) Kashefolgheta, S.; Vila Verde, A. Developing Force Fields When Experimental Data Is Sparse: AMBER/GAFF-Compatible Parameters for Inorganic and Alkyl Oxoanions. *Phys. Chem. Chem. Phys.* **2017**, *19*, 20593–20607.

(4) Michaud- Agrawal, N.; Denning, E. J.; Woolf, T. B.; Beckstein, O. MDAnalysis: A Toolkit for the Analysis of Molecular Dynamics Simulations. *J. Comput. Chem.* **2011**, *32*, 2319–2327.

(5) Fiedler, J.; Thiyam, P.; Kurumbail, A.; Burger, F. A.; Walter, M.; Persson, C.; Brevik, I.; Parsons, D. F.; Boström, M.; Buhmann, S. Y. Effective Polarizability Models. *J. Phys. Chem. A* **2017**, *121*, 9742–9751.

(6) Bergström, S.; Olofsson, G. Thermodynamic Quantities for the Acid Dissociation of Alkylammonium Ions in Water over a Wide Temperature Range. *J. Solution Chem.* **1978**, *7*, 497–513.
